# Mechanisms of Transforming DNA Uptake to the Periplasm of Bacillus subtilis

**DOI:** 10.1128/mBio.01061-21

**Published:** 2021-06-15

**Authors:** Jeanette Hahn, Micaela DeSantis, David Dubnau

**Affiliations:** a Public Health Research Institute, Rutgers University, Newark, New Jersey, USA; b Department of Microbiology, Biochemistry and Molecular Genetics, New Jersey Medical School, Rutgers University, Newark, New Jersey, USA; University of Illinois at Chicago

**Keywords:** *Bacillus subtilis*, ComEA, ComEC, DNA uptake, periplasm, transformation

## Abstract

We demonstrate here that the acquisition of DNase resistance by transforming DNA, often assumed to indicate transport to the cytoplasm, reflects uptake to the periplasm, requiring a reevaluation of conclusions about the roles of several proteins in transformation. The new evidence suggests that the transformation pilus is needed for DNA binding to the cell surface near the cell poles and for the initiation of uptake. The cellular distribution of the membrane-anchored ComEA of Bacillus subtilis does not dramatically change during DNA uptake as does the unanchored ComEA of *Vibrio* and *Neisseria*. Instead, our evidence suggests that ComEA stabilizes the attachment of transforming DNA at localized regions in the periplasm and then mediates uptake, probably by a Brownian ratchet mechanism. Following that, the DNA is transferred to periplasmic portions of the channel protein ComEC, which plays a previously unsuspected role in uptake to the periplasm. We show that the transformation endonuclease NucA also facilitates uptake to the periplasm and that the previously demonstrated role of ComFA in the acquisition of DNase resistance derives from the instability of ComGA when ComFA is deleted. These results prompt a new understanding of the early stages of DNA uptake for transformation.

## INTRODUCTION

Transformation, the ability to internalize high molecular weight environmental DNA, is widespread among bacteria and is a major mechanism of horizontal gene transfer (reviewed in references [Bibr B1][Bibr B2][Bibr B3]). Several proteins that mediate transformation are widely conserved, suggesting that the core mechanism is of ancient origin. Although most of these proteins were identified more than 3 decades ago in a genetic screen (4, 5), much remains to be learned of the mechanisms that mediate the transfer of DNA across the multiple layers that comprise the bacterial surface barrier.

In the Gram-negative bacteria Vibrio cholerae and Neisseria gonorrhoeae, a type 4 pilus (t4 pilus) snares transforming DNA (tDNA) and retracts, threading the tDNA through a pore in the outer membrane formed by a ring of secretin subunits and thus into the periplasm (6–9). There, the tDNA encounters the DNA binding protein ComEA, a periplasmic protein that serves as a Brownian ratchet (10), preventing back diffusion of the tDNA which is believed to accumulate in the periplasm before crossing the inner cell membrane (7–9, 11, 12). In these bacteria, ComEA diffuses to the site of transforming tDNA uptake where it concentrates dramatically as it binds to inward-diffusing tDNA.

Gram-positive bacteria lack an outer membrane. Instead, Bacillus subtilis, the subject of the present study, is surrounded by a formidable cell wall, roughly 30 to 35 nm in thickness, enclosing a 20- to 40-nm-thick periplasm, defined as the compartment between the wall and the cell membrane (13–15). This compartment is probably gel-like, containing proteins, small molecules, and membrane-anchored lipoteichoic acid (14, 16), all of which may restrict diffusion by molecular crowding. The wall of B. subtilis is a complex structure, including peptidoglycan, proteins, and wall teichoic acid (14). These differences in the outer layers of Gram-positive and Gram-negative bacteria suggest consequent differences in their mechanisms of DNA uptake to the periplasm. In fact, wall teichoic acid, which is exclusive to the Gram-positive bacteria, has been proposed to play a role in DNA binding to competent cells (17). Also suggestive of such differences is that B. subtilis ComEA is an integral membrane protein with its C-terminal DNA binding motifs exposed to the periplasm (18, 19) as opposed to the unanchored ComEA proteins of V. cholerae and N. gonorrhoeae that can diffuse within the periplasm.

Transformable Gram-positive bacteria, like their Gram-negative counterparts, encode t4 pilus-related proteins that form filamentous structures. In Streptococcus pneumoniae, these proteins assemble fibers that can extend several micrometers into the extracellular environment and can bind DNA (20–22). No such extended structure has been reported in B. subtilis, which instead seems to form a shorter “pseudopilus” that is probably long enough to traverse the wall (23). This transformation pseudopilus (tpilus) is encoded by the seven genes of the *comG* operon and requires *comC* and *bdbDC* for its construction (23–28). ComC is a membrane peptidase that processes ComGC, the major prepilin subunit as well as several minor prepilins, while BdbD and BdbC are thiol-disulfide oxidoreductases that introduce intramolecular and intermolecular disulfide bonds into ComGC and ComEC, the latter a component of the transformation permease (29). *comGA*, the first gene of the *comG* operon, encodes a traffic ATPase that is required for tpilus assembly (30). By analogy with V. cholerae (6), it is likely that the Gram-positive tpili retract to bring DNA into the periplasmic compartment. Indeed, a recent study shows convincingly that the pili of S. pneumoniae extend and rapidly retract (22), although no such evidence has been reported for B. subtilis.

**FIG 1:**
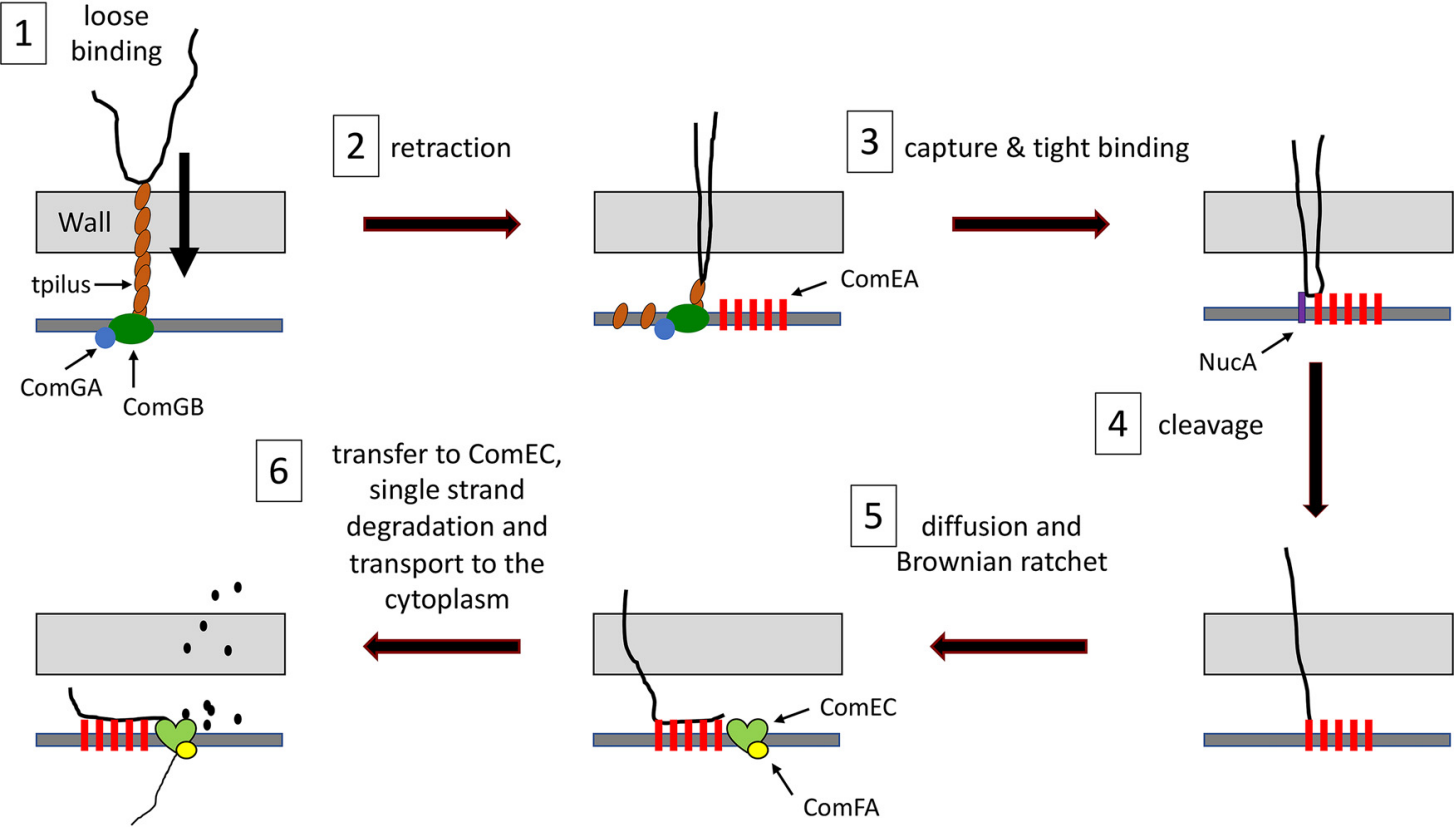
Transformation model. The process is divided into six steps. Each protein is depicted only before the step in which it is required. (Step 1) The transforming DNA binds reversibly to the transforming DNA (tDNA), which extends across the cell wall. Assembly of the tpilus requires the ATPase ComGA. The tpilus is presumably anchored by the ComGB scaffold protein. (Step 2) The tpilus retracts, bringing a loop of DNA into the periplasm. (Step 3) The DNA is captured by ComEA, a membrane-anchored DNA binding protein, and association of the DNA with the cell is stabilized. (Step 4) The membrane DNase NucA cleaves the DNA, providing a terminus for further steps. (Step 5) More DNA diffuses into the periplasm and is captured by additional nearby molecules of ComEA, preventing retrograde diffusion (a Brownian ratchet). (Step 6) DNA is transferred to periplasmic domains of the channel protein ComEC. One strand is degraded, and the other is transported through the channel with the help of the ATPase ComFA.

In all transformable bacteria, transport across the cell membrane is accomplished with the participation of ComEC, which is believed to form a channel for the passage of DNA. In B. subtilis and S. pneumoniae, transport also requires the membrane-associated ComFA ATPase and ComFC, its binding partner (31–35). During transport, one strand of the transforming tDNA is degraded, while the transforming strand enters the cytosol (36), where it associates with DNA binding proteins and recombines with homologous DNA sequences to yield a transformant. A cartoon representation of the transformation process in B. subtilis is shown in Fig. 1, which incorporates results from the present study as well as information from past studies.

In both Gram-negative and Gram-positive bacteria, tDNA becomes resistant to added DNase I (hereafter DNase) during an early step in transformation. In the Gram-negative bacteria, DNase resistance signals that the tDNA has breached the outer membrane. In Gram-positive bacteria, the proper interpretation of DNase resistance is less clear and has been equated with “uptake” or “internalization” (37–39) without precise definition, although it is often assumed to indicate transport to the cytoplasm. The latter assumption was reasonably suggested by the observations that ComFA and ComEC, both required for the acquisition of DNase resistance, are almost certainly needed for transport to the cytoplasm, based on their membrane localizations and molecular properties (Fig. 1).

This study elucidates the process of tDNA uptake into the periplasm of B. subtilis. We show here that DNase resistance reflects entry of tDNA to the periplasm, a step that we refer to as “uptake” to distinguish it from “transport” across the cell membrane. With this understanding, we have visualized the process of uptake using fluorescently labeled tDNA, enabling a reevaluation of the roles of several transformation proteins. On the basis of our results, we present an updated understanding of the early steps in transformation of B. subtilis, which may be applicable to other transformable *Firmicutes*.

## RESULTS

### Experimental design.

To visualize uptake, bacteriophage lambda DNA (48.5 kbp) was labeled using the Label-IT Rhodamine TM reagent (Mirus Bio) that covalently couples the fluorophore predominantly to the N7 position of guanine through a flexible linker, without disrupting Watson-Crick base pairing. To minimize perturbation of the rhodamine-labeled DNA (rDNA) structure, we used a labeling density that modifies fewer than 1% of the bases.

In domesticated strains of B. subtilis, competence for transformation is expressed in only 10 to 20% of the cells. To identify competent cells, the promoter of *comG* was fused at the *amyE* locus to either yellow fluorescent protein (YFP) or cyan fluorescent protein (CFP), which delineate the cytosolic compartments of the competent cells. These isogenic strains behaved identically in our assays. For each experiment, aliquots of YFP- and CFP-expressing competent cells were usually combined and then incubated with rDNA, fixed with paraformaldehyde to stop transformation, and then visualized by epifluorescence and phase-contrast microscopy. Depending on the experiment, the YFP- and CFP-expressing samples were derived from wild-type and mutant strains or from samples incubated with and without DNase after transformation. In the latter case, the samples were combined after fixation. This permitted direct comparisons without differences due to image processing and the vagaries of agarose pads.

### DNase as a tool for transformation studies.

Studies in B. subtilis and S. pneumoniae have used 50 to 200 μg/ml of DNase under various conditions to investigate transformation (4, 19, 34, 37, 38, 40). We sought to standardize a DNase treatment that would remove attached rDNA from the surfaces of the cells, without degrading rDNA that had entered the unidentified protected compartment. Preliminary experiments comparing treatment with DNase concentrations from 10 to 100 μg/ml after 15 min of incubation with rDNA, when the acquisition of DNase resistance has reached about half its maximum level (37), showed no differences in the intensities of the rhodamine signals after DNase treatment, comparing the smallest and largest amounts of DNase. If DNase significantly penetrated the protected compartment, we would have expected to see a dose-dependent decrease in the residual rDNA signal. We therefore settled on a 3-min incubation with 100 μg/ml of DNase at 37°C.

**FIG 2:**
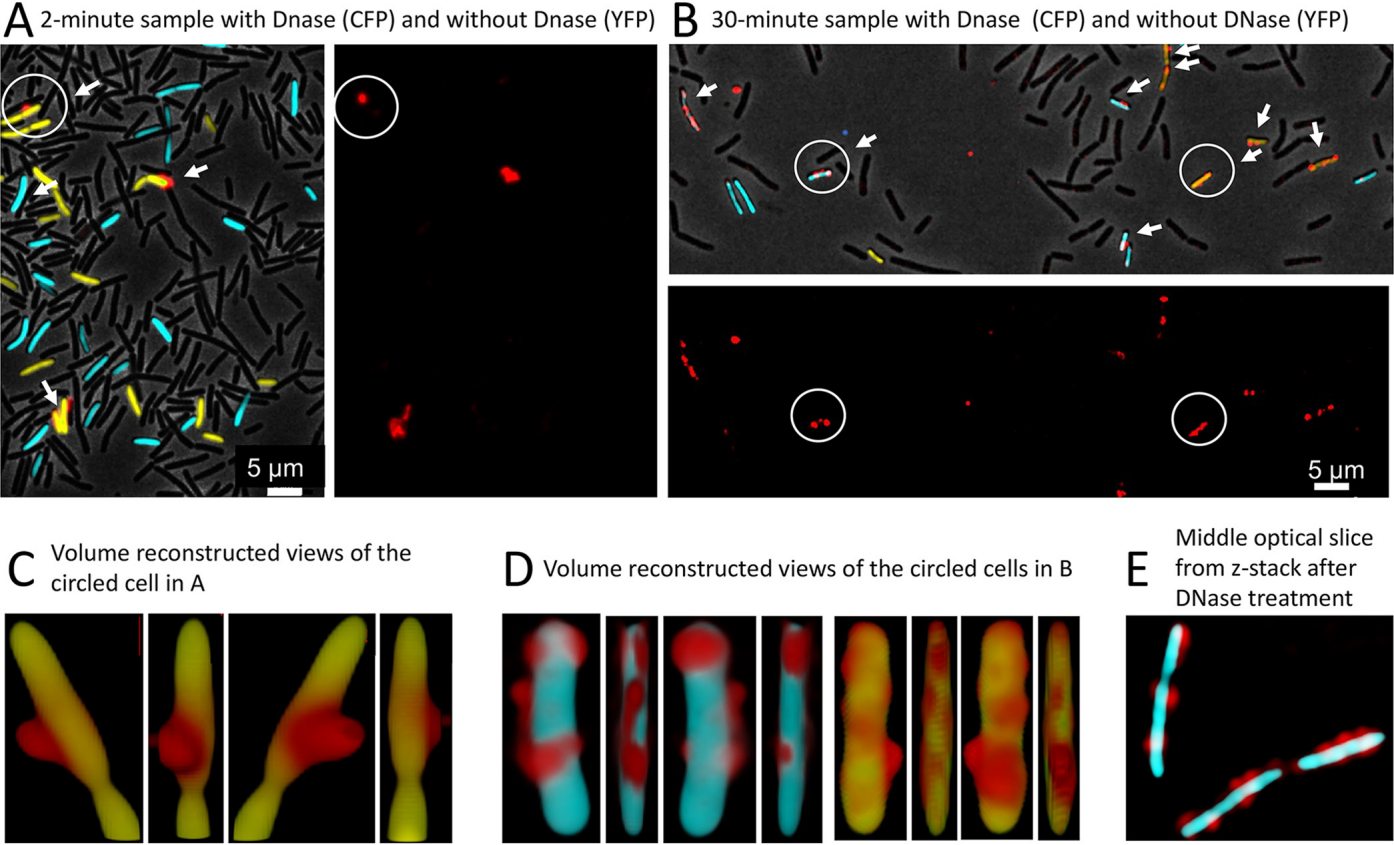
Binding of tDNA and acquisition of DNase I (DNase) resistance. Competent cells expressing CFP (BD5810) or YFP (BD6011) from the *comG* promoter were incubated separately with rDNA, and samples were taken after 2 (A) and 30 (B) min of incubation. The CFP samples were treated with DNase and then combined with YFP samples from the same time points after removing the DNase. In panels A and B, images are presented with superimposed rhodamine, CFP, YFP, and phase-contrast channels as well as one showing only the rhodamine channel. Cells associated with rDNA are indicated by arrows. The circled cell in panel A was imaged by 3D deconvolution and volume reconstructed, and views produced by successive 90° rotations are shown in panel C. In panel D, volume-reconstructed images are shown for the single cyan (with DNase) and yellow (no DNase) cells, circled in panel B. Panel E contains a 3D-deconvolved optical slice from the center of a Z-stack, showing of a group of cells after DNase treatment.

Wild-type cultures expressing YFP or CFP were each incubated with 0.2 μg/ml rDNA, close to a saturation concentration for transformation. The CFP samples were treated with DNase and were then mixed with untreated YFP cells from the same time points and visualized for fluorescence in the YFP, CFP, and rhodamine channels as well as by phase-contrast microscopy after 2- and 30-min incubation with rDNA. In the 2-min sample (Fig. 2A), about 4% of the competent cells and fewer than 1% of the noncompetent cells were associated with rDNA, and after DNase treatment, no cells were detected that retained a significant rhodamine signal. After 30-min incubation (Fig. 2B), many of the competent cells showed an rDNA signal without DNase treatment, while again fewer than 1% of the noncompetent cells were associated with rDNA, showing that association is biologically relevant. In several experiments, 55 to 83% of the competence-expressing cells showed association with rDNA after 30 min of incubation (Table 1). After DNase treatment, many of the cells from the 30-min sample were still associated with rDNA (Fig. 2B). In repeated experiments, this number varied from 29 to 85% (Table 1), underscoring the importance of including internal wild-type cell controls in each mutant comparison, as described below. The data presented to this point, suggest that rDNA first binds specifically to the surfaces of competent cells where it is DNase sensitive and later becomes DNase resistant by entering a protected compartment. DNase resistance is largely complete at 30 min, consistent with previous observations using radiolabeled tDNA (37), suggesting that the uptake of rDNA is not markedly impeded by its covalent adducts.

**TABLE 1:** Binding and uptake of rDNA by transformation mutants^*a*^

Strain	No DNase treatment				With DNase treatment			
	With signal	*N*	% with signal	*P* value^*b*^	With signal	*N*	% with signal	*P* value
*ΔcomEA*	12	99	12.1		2	133	1.5	
Wild-type	82	99	82.8	<0.001	92	108	85.2	<0.001

*ΔnucA*	39	137	28.5		13	126	10.3	
Wild-type	88	138	63.8	<0.001	70	123	56.9	<0.001

*nucA D98A*	45	114	39.5		21	126	16.7	
Wild-type	70	124	56.5	<0.009	47	162	29.0	0.015

*ΔcomFA*	45	110	40.9		32	104	30.8	
Wild-type	86	110	78.2	<0.001	80	103	77.7	<0.001

*comFA K152E*	82	136	60.3		91	163	55.8	
Wild-type	65	118	55.1	n.s.	68	103	66.0	n.s.

*comEC518*	66	107	61.7		47	150	31.3	
Wild-type	112	147	76.2	0.01	91	136	66.9	<0.001

a Data for each mutant were obtained in mixed suspension with a wild-type control.

b Determined with a two-tailed z-test. n.s., not significant.

### rDNA enters the periplasm where it becomes resistant to added DNase.

It is likely that rDNA cannot cross the cell membrane because of its bulky adducts (rhodamine plus a positively charged linker) and therefore accumulates in the periplasm. Although we have found that rDNA with a labeling density like that used in this study is reduced in its ability to transform, two considerations make it impossible to interpret this reduced transforming capacity. First, low density random labeling will leave variable stretches of label-free DNA that may be capable of transforming. On the other hand, transformation could be blocked by the adducts at the level of recombination, gene expression, or replication.

To investigate the location of DNase-resistant rDNA further, we generated three-dimensional (3D)-deconvolved, volume-reconstructed images of transformed CFP- and YFP-labeled competent cells with and without DNase treatment (Fig. 2C and D). The images in Fig. 2 show representative cells, viewed from four directions, related by 90° rotations. The three images in Fig. 2C show a typical cell not treated with DNase and imaged after 2 min of incubation with rDNA, a time when no tDNA is protected from DNase, as noted above. The projected views show a blob of rDNA, exhibiting limited contact with the surface of the YFP volume. λ DNA molecules in solution are coils with an average radius of gyration equal to about 500 nm (41). Figure 2C is consistent with binding of such an rDNA coil to the cell surface, involving a limited portion of the rDNA surface. In cells imaged after 30 min of incubation, whether DNase treated or not, the rDNA is more closely juxtaposed to the surface (Fig. 2D). In Fig. 2D, some of the rhodamine signal is stretched across the surface, and comparison of the rotated views shows rDNA that appears to wrap around the cell volume, consistent with its location within the confined space of the periplasm and its extension across the outer face of the cell membrane. Figure 2E shows a single optical slice through three cells, imaged after DNase treatment following 30-min incubation with rDNA, confirming that the rDNA signal is localized at the edges of the cells, consistent with a periplasmic location and with the failure of rDNA to cross the membrane. Figure 2C to E thus suggest that rDNA first associates with the wall surface by localized contact and then enters the periplasmic compartment where it becomes DNase resistant. Subsequently, rDNA extends across the cell membrane surface (see Fig. 1 in the supplemental material). Although throughout we have used fixation to facilitate accurate sample timing, indistinguishable 3D reconstructed images to those shown in Fig. 2 were obtained without fixation, as shown in Fig. S1. Again, the rDNA adheres to the cell and exhibits spreading across the volume surface.

10.1128/mBio.01061-21.3 FIG S1 Volume reconstructions without fixation. Wild-type competent cells were incubated for 30 min with rDNA, washed, and placed on agarose pads for microscopy. Seven cells are shown, with single viewing angles. Download FIG S1, PDF file, 0.04 MB. Copyright © 2021 Hahn et al. 2021 Hahn et al. https://creativecommons.org/licenses/by/4.0/ This content is distributed under the terms of the Creative Commons Attribution 4.0 International license.

### ComFA stabilizes ComGA.

Before studying the roles of individual proteins in rDNA uptake, we determined the impact of each deletion mutation on the stabilities of other transformation proteins. Figure S2 shows Western blots in which we have examined *ΔcomFA*, *ΔcomGA*, *ΔnucA*, *ΔcomC*, *ΔcomEA*, and *comEC518* mutant extracts using antisera raised against ComGA, ComFA, NucA, ComEA, and ComEC. All the mutations are deletions except that *comEC518* is a Tn*917* insertion, which exhibits no transformability and is located near the start of the *comEC* coding sequence after residue V50. All of the proteins are present at close to normal levels in the mutants, with the important exception of ComGA, which is destabilized in the absence of ComFA (Fig. S2B), as reported previously (42), suggesting that ComGA and ComFA are binding partners. ComGA is present at the normal level in an inactivating K152E mutant of *comFA* (33, 35). K152 lies in the Walker A motif of ComFA, demonstrating that the ATPase activity of ComFA is not needed for ComGA stability. In fact, ComGA interacts with both ComFA and ComFC (43), and the uptake-associated *ΔcomFA* phenotype may be due to an indirect effect on ComGA

10.1128/mBio.01061-21.4 FIG S2 Western blots for transformation proteins in mutant strains. All of the mutants were deletions except for *comEC518* which is a transposon insertion near the beginning of the coding sequence. In all cases, the relevant knockout mutant was used as a negative control for the antiserum. For most of the gels, a loading control is shown using anti-elongation factor G (EFG) antiserum, a kind gift from J. Dworkin. In panel D, the cross-reacting band just below the ComEA signal serves as a loading control. There is a consistent (29) cross-reacting band at the position of the ComEC signal, evident in the *comEC518* lanes in panel E. Download FIG S2, PDF file, 0.3 MB. Copyright © 2021 Hahn et al. 2021 Hahn et al. https://creativecommons.org/licenses/by/4.0/ This content is distributed under the terms of the Creative Commons Attribution 4.0 International license.

### Testing mutants for rDNA binding and uptake.

We next examined mixtures of YFP- and CFP-labeled wild-type and mutant strains. To analyze the raw images, we utilized several tools. For each mutant, we counted the percentage of competent cells for mutant and wild-type strains that were associated with detectable rDNA signals. Second, we measured the signal intensities in the rDNA (rhodamine) channel for those cells that did exhibit fluorescence after DNase treatment. These two types of quantitation provided a more complete understanding than either measurement alone. Additionally, we performed volume reconstructions for several of the mutants. Although the frequencies of cells associated with rDNA (Table 1) and rDNA image intensities (Table 2) could be quantified, the distribution of rDNA within the cells is difficult to quantify, and therefore, we present a series of images for each mutant.

**TABLE 2:** Quantitation of uptake (normalized fluorescence intensities)

Mutant gene	Wild-type		Mutant		*P* value^*a*^
	*N*	Mean intensity^*b*^ ± SD	*N*	Mean intensity ± SD	
*comFA K152E*	89	2,515 ± 2,270	124	2,591 ± 2,588	n.s.
*ΔnucA*	177	3,396 ± 4,449	40	1,197 ± 2,325	<0.001
					<0.001
*nucA D98A*	99	2,789 ± 3,716	55	1,523 ± 2,454	0.012
					0.002
*comEC518*	127	5,412 ± 4,247	73	1,517 ± 1,199	<0.001
					<0.001

a For each mutant, the top *P* value was determined with a two-tailed Z-test, and the bottom *P* value was determined with a Mann-Whitney U-test. n.s., not significant.

b Fluorescence intensities (in arbitrary units of wild-type and mutant cells that exhibited rDNA fluorescence) were measured and averaged. Comparison of wild-type intensities in different fields yielded normalization factors that were used to combine the data from separate fields for a given mutant.

The *ΔcomGA* and *ΔcomC* mutants exhibited no detectable association with rDNA even without DNase treatment (Fig. S3). Because ComGA is needed to assemble tpili and ComC is a peptidase that processes the major and minor pilins for assembly (23), these results support the hypothesis that tDNA binding requires tpili (Fig. 1), most likely because the DNA binds directly to these organelles, as it does in S. pneumoniae (20) and in V. cholerae (6).

10.1128/mBio.01061-21.5 FIG S3 DNA binding in *ΔcomGA* and *ΔcomC* strains. *ΔcomGA* (A) and *ΔcomC* (B) strains expressing CFP were combined with wild-type bacteria expressing YFP. Transformation with rDNA was for 30 minutes without DNase treatment. The arrows indicate all the cells with detectable rDNA signals. The CFP-labeled *ΔcomGA* cells are slightly filamented (43). Download FIG S3, PDF file, 0.1 MB. Copyright © 2021 Hahn et al. 2021 Hahn et al. https://creativecommons.org/licenses/by/4.0/ This content is distributed under the terms of the Creative Commons Attribution 4.0 International license.

**FIG 3:**
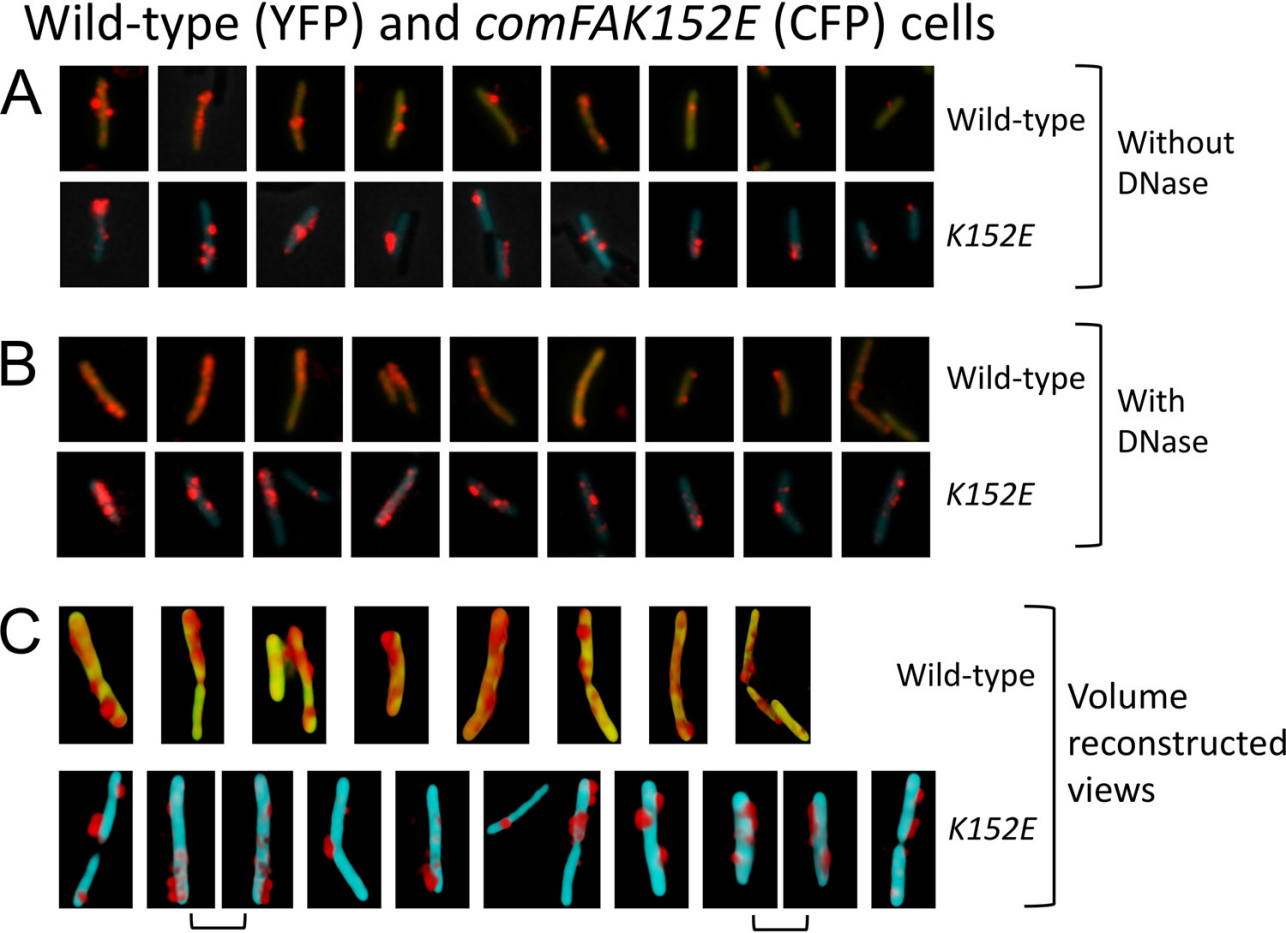
Binding and uptake in the *comFA* K152A mutant. Wild-type (YFP) and mutant (CFP) cells were combined and then incubated for 30 min with rDNA. Panels A and B show results for *comFA K152E* without and with DNase treatment, respectively. In each panel, the top and bottom rows show nine each of wild-type and mutant cells, respectively. All the cells were taken from a single representative microscope field and show all or nearly all of the rDNA-associated cells in that field. In the cropped images of a given panel (A or B), rDNA signals were enhanced identically but rDNA signal intensities cannot be compared between panels A and B. Panel C shows volume-reconstructed images of several representative wild-type (top row) and mutant (bottom row) cells after DNase treatment. In each case, a single view is shown except for the adjacent images in panel C joined by brackets, which show two views of the same cell related by 180^°^ rotations.

ComFA is a membrane-localized ATPase that is predicted to assist in the transport of tDNA to the cytoplasm (Fig. 1). In the *ΔcomFA* strain, there is a significant decrease in the percentage of cells that were associated with rDNA after 30 min, either with or without DNase treatment (Table 1). Despite this, many *ΔcomFA* cells did bind and take up rDNA. In the absence of DNase treatment, the signal intensities of the wild-type and mutant cells were similar (Fig. S4A), but with treatment, the signals in the mutant were obviously weaker (Fig. S4B), in agreement with results obtained using radiolabeled tDNA (32, 34). If the decrease in signal intensity in the *ΔcomFA* strain is due to a deficiency in ComGA (Fig. S2B), we would expect the *comFA K152E* mutant to behave like the wild type. Indeed, no significant effect of the K152E mutant was observed on the percentage of cells showing rDNA fluorescence either with or without DNase treatment (Table 1). The images in Fig. 3A and B and the measured intensity values in Table 2 show no effects of this mutation on either the total or DNase-resistant signal intensities, confirming that the effect of *ΔcomFA* on the acquisition of DNase resistance and on rDNA binding to the cells is due to ComGA deficiency. In Fig. 3 and subsequent figures, there is evident cell-to-cell heterogeneity in the amounts of tDNA associated with the cells for both the wild-type and mutant cells. This is also reflected in the large standard deviations of the measurements in Tables 1 and 2. This is likely due to asynchrony in tDNA binding to the cells, in the on-going development and loss of transformability, and probably also to heterogeneity in the expression of transformation proteins at peak competence.

10.1128/mBio.01061-21.6 FIG S4 Binding and uptake in the *ΔcomFA* mutant. Wild-type (YFP) and mutant (CFP) cells were combined before 30-minutes incubation with rDNA. Panels A and B show results without and with DNase treatment, respectively. In each panel, the top and bottom rows show nine each of wild-type and mutant cells, respectively. All the cells were taken from a single representative microscope field and show all or nearly all of the rDNA-associated cells in that field. In the cropped images of a given panel, rDNA signals were enhanced identically, but rDNA signal intensities cannot be compared between panels A and B. Download FIG S4, PDF file, 0.1 MB. Copyright © 2021 Hahn et al. 2021 Hahn et al. https://creativecommons.org/licenses/by/4.0/ This content is distributed under the terms of the Creative Commons Attribution 4.0 International license.

Despite the nonrequirement of ComFA for uptake, the rDNA signal in the K152E mutant cells after DNase treatment is not spread out across the cell surface as it is in the wild-type cells, suggesting an unexpected ATP-dependent requirement for ComFA in the spreading of the rDNA in the periplasm (Fig. 3C).

**FIG 4:**
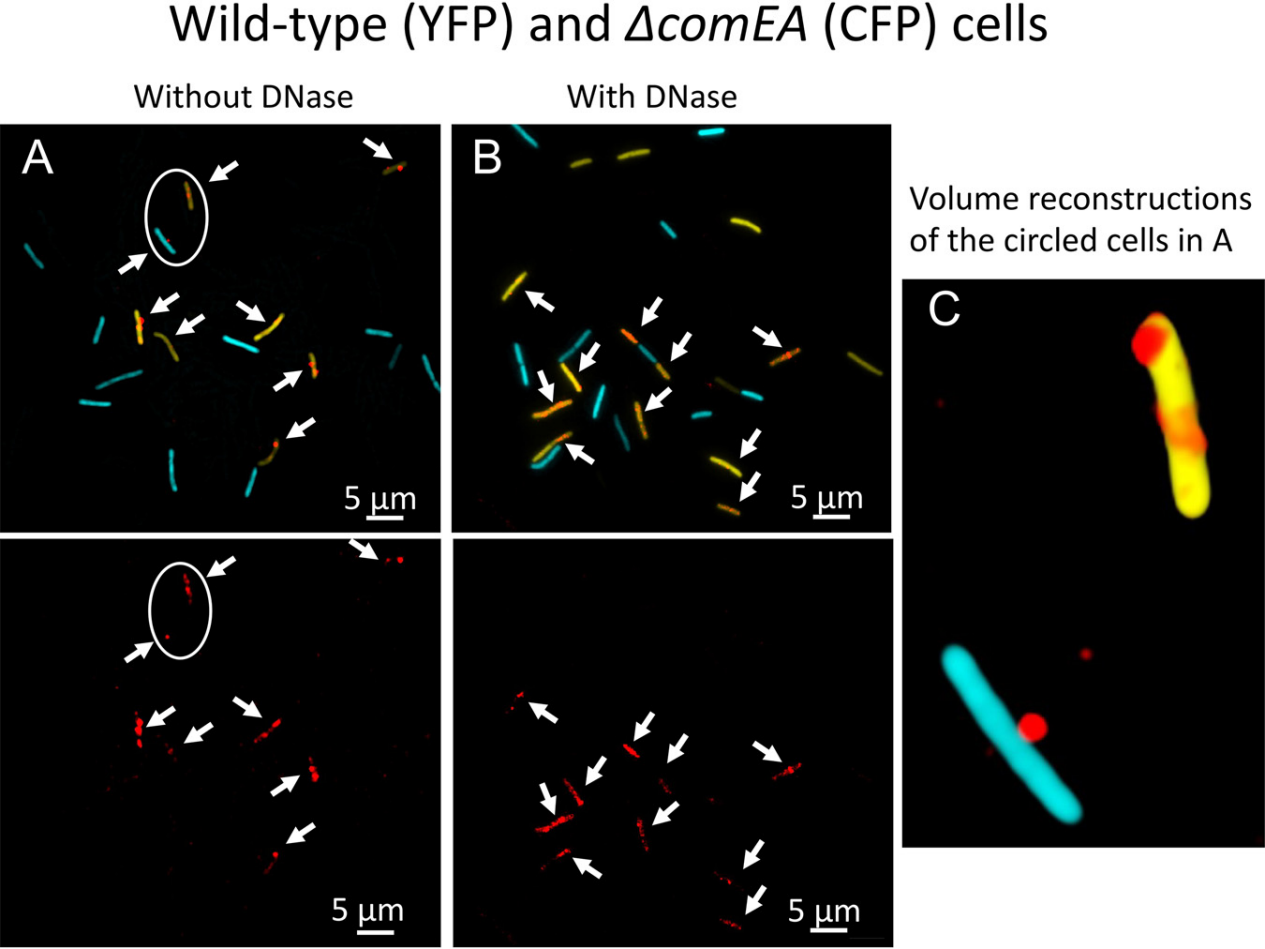
Binding and uptake in the *ΔcomEA* mutant. Wild-type (YFP) and *ΔcomEA* cells were combined before incubation for 30 min with rDNA. Panel A shows the cells without DNase treatment, and panel B shows cells with treatment. In the top images, the rhodamine, CFP, and YFP channels were merged, and the bottom images in panels A and B show only the rhodamine channel. The arrows indicate the positions of all the rhodamine signals. Panel C shows one aspect from a 3D deconvolution of the CFP- and YFP-expressing cells circled in panel A.

ComEA is a membrane-localized DNA binding domain with its binding domain presented in the periplasm (Fig. 1). In a *ΔcomEA* strain, the percentage of cells with associated rDNA signals was reduced about 7-fold in the absence of DNase treatment (Fig. 4A and Table 1), and the frequency of cells with DNase-resistant rDNA was reduced 57-fold (Fig. 4B and Table 1), consistent with results obtained with B. subtilis and S. pneumoniae using radiolabeled DNA (19, 37, 38). In the *ΔcomEA* cells that did exhibit binding, the rDNA remained on the cell surface (Fig. 4C), like the wild-type image at 2 min (Fig. 2A). Thus, ComEA assists in binding and is required for uptake. Most likely, the initial binding of tDNA to the tpilus is labile and must be stabilized by contact with periplasmic ComEA, a known DNA binding protein. Indeed, reversible attachment of DNA to the surface of B. subtilis competent cells has been reported (44). The nearly total DNase susceptibility of rDNA in the mutant demonstrates that ComEA is also needed for uptake to the periplasm.

**FIG 5:**
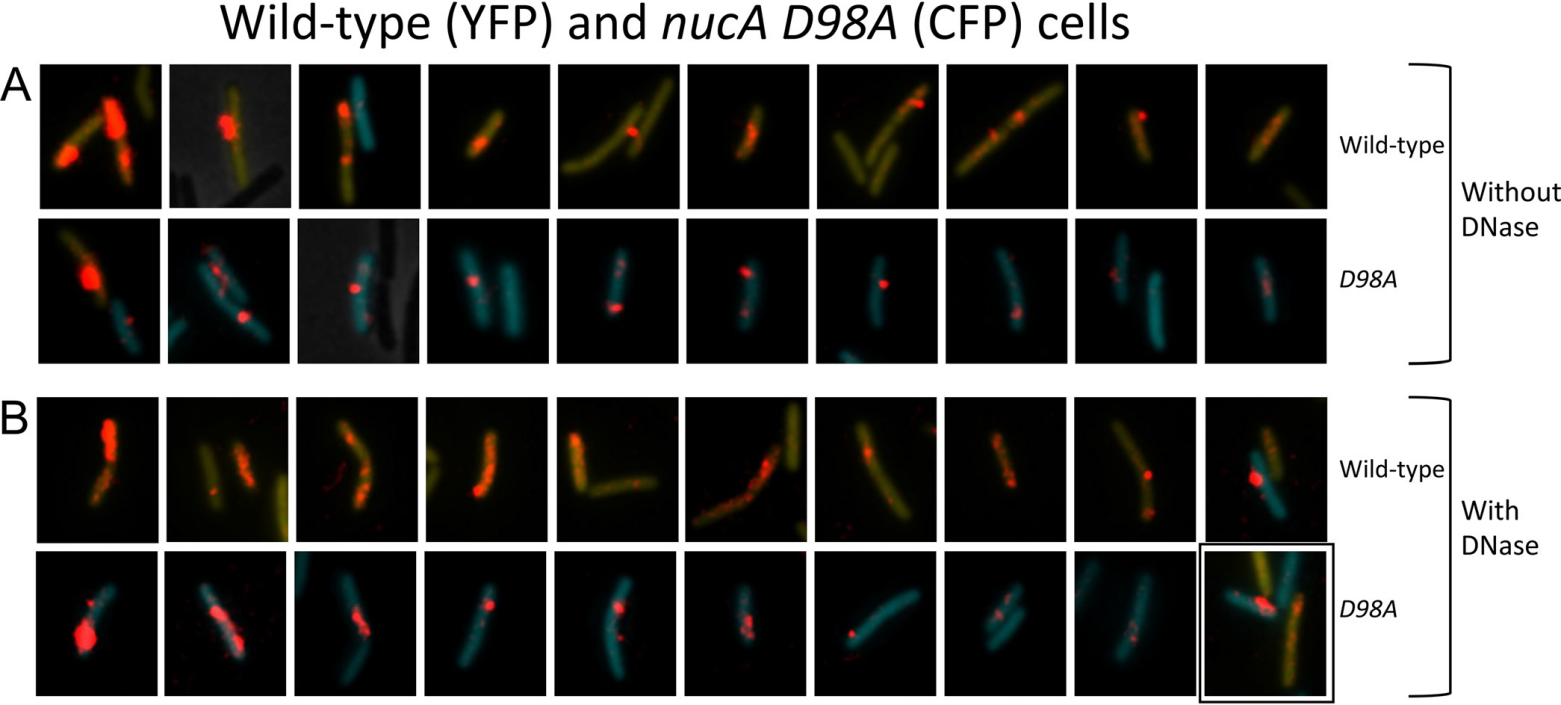
Binding and uptake in the *nucA* D98A mutant. (A and B) Wild-type (YFP) and D98A (CFP) mutant cells were made competent, combined, and after 15-min incubation with rDNA, imaged without (A) and with DNase (pB). In each of these panels, the top and bottom rows show nearly all the wild-type and mutant cells with rDNA signals from a single field. The rhodamine signal was enhanced before cropping the cells, and the intensities of the rhodamine signal can be directly compared within the panels. The YFP and CFP images were separately adjusted in each image so as not to obscure the rDNA signal. The images in these two panels were ordered by apparent rDNA signal strengths, decreasing from left to right, to facilitate comparisons of the mutant and wild-type cells. The boxed image in panel B shows a cell expressing both YFP and CFP.

NucA is an endonuclease that is attached to the membrane by its N-terminal transmembrane helix, while its C-terminal nuclease domain extends into the periplasm (37) (Fig. 1). A *ΔnucA* strain exhibits decreased rates of transformation, tDNA binding, and the acquisition of DNase resistance (37). To explore this phenotype with rDNA, we used samples incubated with rDNA for only 15 min to maximize the differences between the mutant and wild-type strains, because the final yield of transformants in the mutant is reduced only twofold (37). The percentage of *ΔnucA* cells associated with rDNA after DNase treatment (Table 1) was reduced 5.5-fold, and the rDNA signal intensities were reduced 2.8-fold after DNase treatment (Table 2) for a combined 15-fold effect on uptake. This decrease in signal intensity is evident in the images shown in Fig. S5. To determine whether the uptake deficiency of the *ΔnucA* strain was due to the nuclease activity of NucA, we inserted a D98A active site mutation at the native *nucA* locus. The design of this mutation was based on analysis of NucB (45), which exhibits 58% identity with NucA. D98A corresponds in position to residue D87A in NucB. This mutant exhibits a transformation deficiency like that of the *ΔnucA* strain. The data in Table 1 show that the D98A mutation, like *ΔnucA*, decreases the frequency of cells associated with rDNA after DNase treatment although to a lesser extent, perhaps due to residual nuclease activity. Figure 5 and Table 2 show that the intensities of the rDNA signals in this mutant were also decreased following DNase treatment, confirming that the nuclease activity of NucA is needed for uptake to the periplasm and excluding a polar effect of *nucA* inactivation on the downstream *nin* gene, which has an independent role in transformation (37, 46). Volume reconstructions after DNase treatment for the *ΔnucA* (Fig. S6A) and D98A (Fig. S6B) mutants show that the rDNA is not spread out as it is in the wild-type, like the phenotype of the *comFA K152E* mutant. Based on the earlier assumption that DNase resistance corresponds to entry to the cytoplasm, it had been proposed that NucA produced ends for transport through the membrane channel (37). Although this is plausible, the nuclease activity also plays a role in uptake to the periplasm.

10.1128/mBio.01061-21.7 FIG S5 Binding and uptake in the *ΔnucA* mutant. Wild-type (YFP) and mutant (CFP) cells were combined before 15-minute incubation with rDNA and imaged without (A) and with (B) DNase. In each of these panels, the top and bottom rows show nearly all the wild-type and mutant cells, respectively, from a single field, that manifest associated rDNA. The rhodamine signal was enhanced before cropping the cells, and the intensities of the rhodamine signal can be directly compared within each panel. The YFP and CFP images were separately adjusted in each image so as not to obscure the rDNA signal. The images in these two panels were ordered by apparent rDNA signal strengths decreasing from left to right to facilitate comparisons of the mutant and wild-type cells. The boxed images each show both a YFP- and a CFP-expressing cell. Download FIG S5, PDF file, 0.1 MB. Copyright © 2021 Hahn et al. 2021 Hahn et al. https://creativecommons.org/licenses/by/4.0/ This content is distributed under the terms of the Creative Commons Attribution 4.0 International license.

10.1128/mBio.01061-21.8 FIG S6 Volume reconstructions of the *ΔnucA* (A) and *nucA* Δ98A (B) mutant strains. Wild-type (YFP-expressing) and mutant (CFP-expressing) cells are shown in each panel for comparison. The lonely panel to the right shows both a wild-type cell and a *ΔnucA* cell. Download FIG S6, PDF file, 0.3 MB. Copyright © 2021 Hahn et al. 2021 Hahn et al. https://creativecommons.org/licenses/by/4.0/ This content is distributed under the terms of the Creative Commons Attribution 4.0 International license.

**FIG 6:**
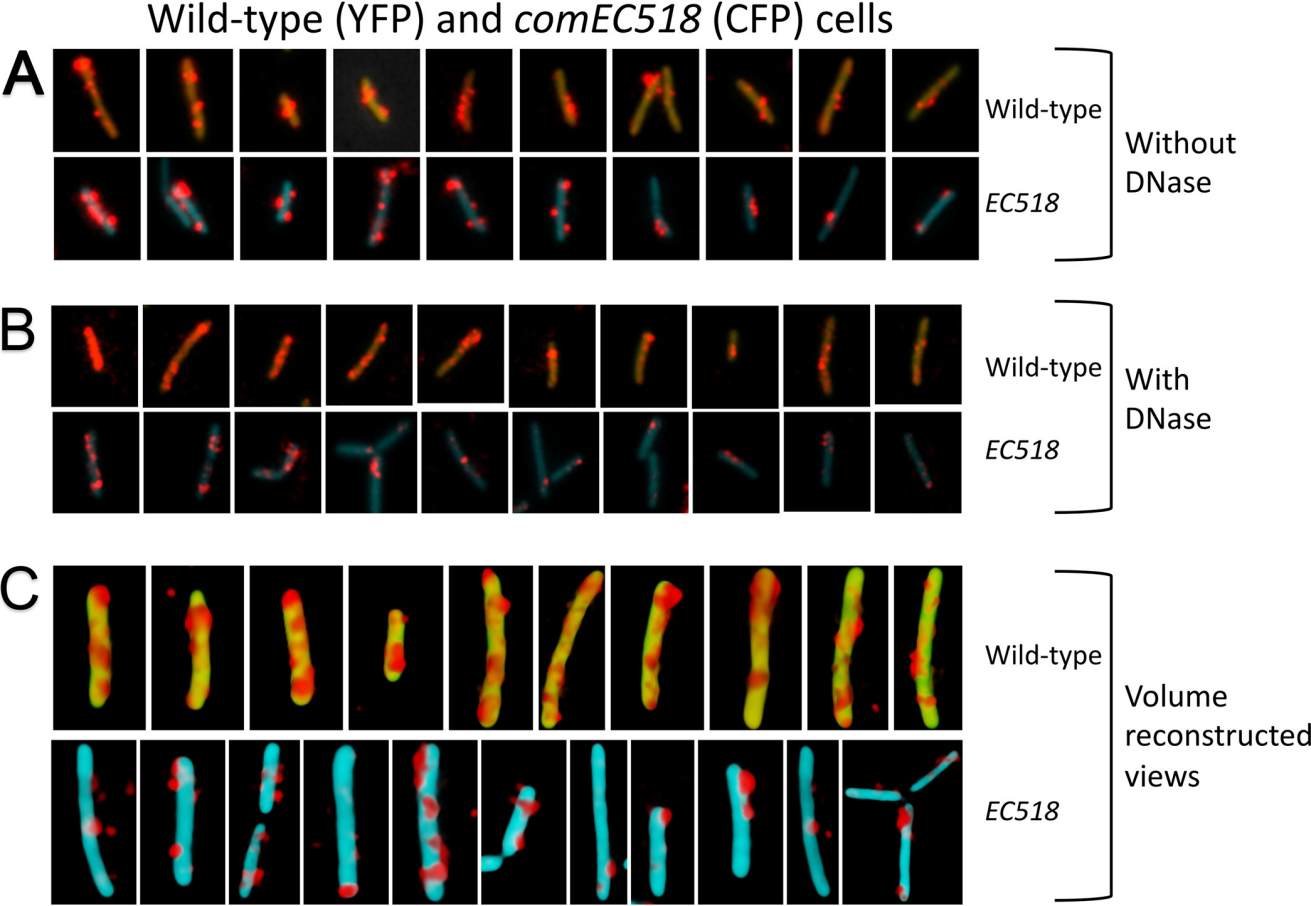
Binding and uptake in the *comEC518* mutant. Wild-type (YFP) and *ΔcomEC518* (CFP) cells were combined before 30-min incubation with rDNA. The images were processed as described in the legend to Fig. 5. Panel A shows the cells without DNase treatment, and panel B shows cells with treatment. The images in these two panels were ordered by apparent rDNA signal strengths decreasing from left to right, to facilitate comparisons of the mutant and wild-type cells. Panel C shows selected volume-reconstructed images of wild-type and *comEC518* cells, DNase treated, selected from the same field, representing almost all the cells in the field. Only one view is shown of each cell. Image intensities cannot be compared either within panel C or with the images in panels A and B.

ComEC is a multipass integral membrane protein that forms a channel for tDNA transport (Fig. 1). As noted above, the use of radiolabeled rDNA showed that the elimination of ComEC did not affect the total association of tDNA with competent cells but markedly reduced the label after DNase treatment (4, 37, 38). The use of rDNA should therefore show a previously unexpected role for ComEC in uptake. After 30 min, the fraction of *comEC518* competent cells showing DNase-resistant rDNA was moderately decreased in the *comEC518* mutant (31%) compared with 67% for the wild type (Table 1), probably a significant difference (*P* = 0.01). In contrast, there is little if any effect on the frequency of rDNA attachment before DNase treatment. This trend is confirmed by the images contained in Fig. 6. Figure 6A shows that in the absence of DNase treatment, the intensities of the rDNA signals are comparable in wild-type and mutant cells. However, Fig. 6B and Table 2 show that the rDNA signal in the DNase-treated *comEC518* cells that exhibit uptake is lower than in the wild type, in agreement with measurements made using radiolabeled tDNA (4, 19, 34, 37). The combined effects of the decreased frequency of DNase-resistant fluorescent cells and the decreased signal intensity would result in nearly an eightfold decrease in uptake to the periplasm. Interestingly, the volume-reconstructed images in Fig. 6C reveal a difference in the distribution of the DNase-resistant rDNA signals in wild-type and *comEC* cells. In the wild-type cells, as noted above (Fig. 2B), rDNA is extended across the volume surface. But in the *comEC518* cells, the DNase-resistant rDNA is not spread, as noted for the *nucA* and *comFA K152E* mutants. Thus, although binding to the cell surface is essentially normal in the *comEC* mutant, uptake is strongly affected, and the reduced amount of rDNA that manages to enter the periplasm is confined locally.

### rDNA closely associates with YFP-ComEA during uptake.

In the Gram-negative systems, periplasmic ComEA is initially distributed uniformly within the periplasm and then accumulates at the sites of DNA uptake (8, 9). To examine the interactions of rDNA with ComEA *in vivo*, we made a YFP-ComEA construct in which the N-terminal YFP moiety was in the cytosol and the DNA binding portion of ComEA was appropriately located in the periplasm. The *yfp-comEA* construct was placed at the *comE* locus, expressed normally under competence control, and the native *comEA* was deleted. The resulting strain, with *yfp-comEA* as the only source of *comEA* in the cell, was normally transformable, although the rate of appearance of DNase-resistant transformants was deceased about twofold. As reported previously using immunofluorescence with native protein (47) and by Kaufenstein et al. (48) with a YFP-ComEA construct similar to ours, ComEA forms large foci at several locations around the membrane. Uniquely among the transformation proteins, there is no obvious preference of these foci for polar or subpolar locations. The foci are not caused by the uptake of DNA from occasional lysed cells, because the addition of DNase (100 μg/ml) during 90 min of growth prior to sampling had no effect on this distribution.

**FIG 7:**
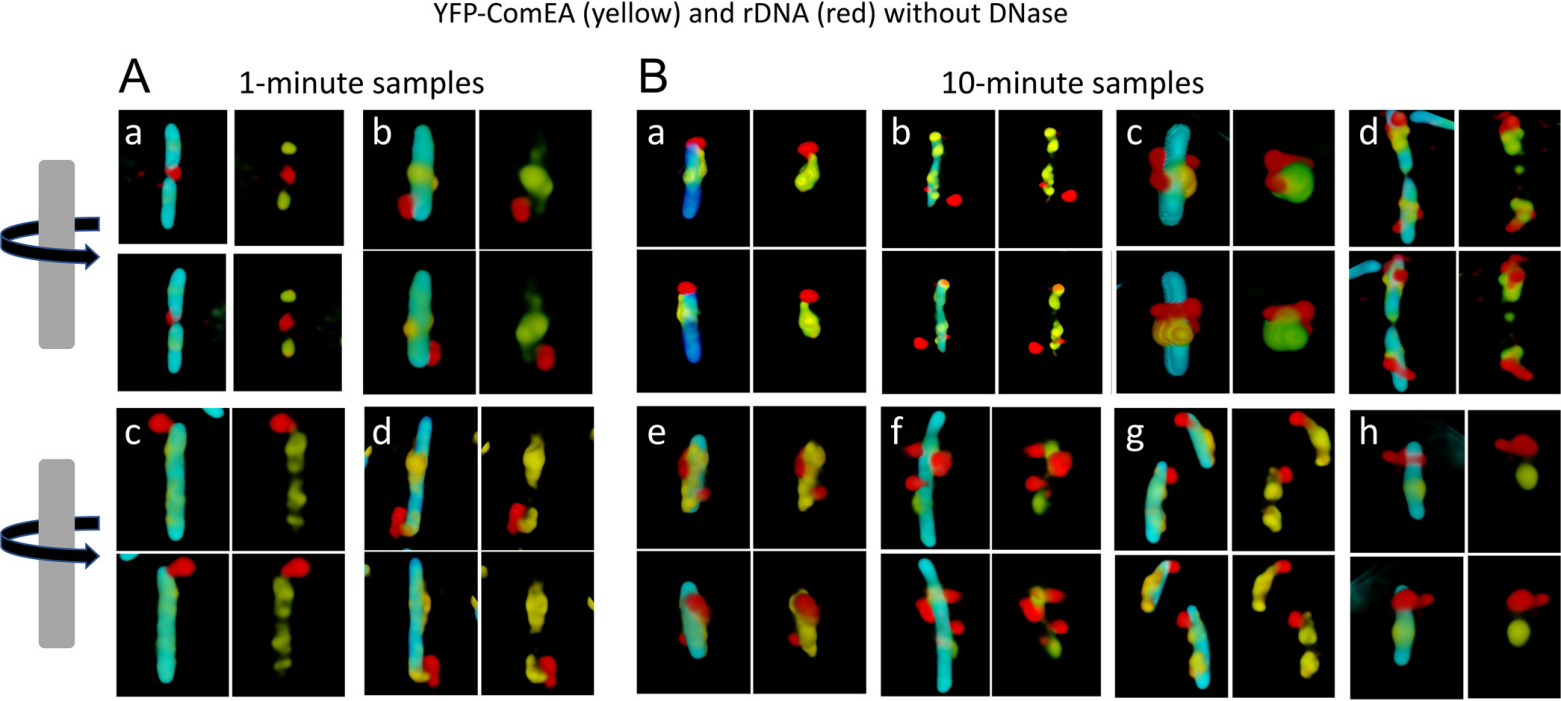
Association of rDNA and YFP-ComEA without DNase treatment. In each group of four images (a to d in panel A and a to h in panel B), the top and bottom pairs show views of a single cell, related by 180° rotations. The left-hand images in each group show the superimposed YFP, rhodamine, and CFP channels, where CFP delineates the cytoplasmic volume. The right-hand images show only the YFP and rhodamine channels. Panel A shows images collected after 1-min incubation of the cells with rDNA and panel B shows images collected after 10 min.

After the addition of rDNA, we could detect no difference in the distribution of YFP-ComEA foci with and without bound rDNA. Figure 7A shows volume-reconstructed images of four representative cells from a single field that exhibit significant rDNA attachment after 1-min incubation with rDNA. In these images, the rhodamine signal is almost always localized near the poles as reported previously using a different DNA labeling protocol (47). The rDNA in Fig. 7A is consistently near a blob of YFP-ComEA in all the cells shown except in panel Aa, which may show a cell with initial attachment to a tpilus that is not located near an accumulation of ComEA. In the remaining cells of panel A, the rDNA and YFP-ComEA appear to be connected by faint regions of YFP signal intensity. After 10 min of incubation, the images are strikingly different (Fig. 7B); the rDNA is often intimately associated with foci of YFP-ComEA and is sometimes located away from the cell poles (Fig. 7B, cells c and d). Also at 10 min in this same experiment, much of the rDNA intensity is resistant to DNase. These images suggest that tDNA initially attaches to tpili at the poles and is then pulled into the periplasm, where it binds to locally mobile molecules of ComEA (Fig. 1). The Graumann laboratory has reported that the diffusive behavior of YFP-ComEA in the membrane is like that of tDNA in the periplasm, suggesting that these molecules are associated (48), consistent with this conclusion. After the capture event, which results in stabilized binding but no major redistribution of ComEA, the tDNA is plausibly pulled into the periplasm by a Brownian ratchet mechanism involving ComEA and ComEC, where it is stretched across clusters of ComEA and ComEC molecules.

## DISCUSSION

The appreciation that DNase resistance is imparted by the cell wall barrier permits the interpretation of data acquired in this study and the reinterpretation of data from the literature. DNase has a calculated molecular mass of 29 kDa, and it has been estimated from *in vitro* studies that globular proteins of more than about 25 kDa cannot diffuse through the cell wall of B. subtilis (49). The wall preparations used to produce that estimate were treated to remove associated proteins, and the intact wall is likely to be even denser and thus more restrictive to diffusion.

The conceptual partition of transformation proteins into three exclusive categories based on their requirements for binding, uptake, and transport does not fit with the new data. Thus, ComEA is needed for both tight binding and uptake. ComGA appears to be needed for both initial binding and for uptake because the *ΔcomFA* mutation, which lowers the amount of ComGA, decreases the frequency of rDNA binding (Table 1) and is deficient in the amount of uptake in individual cells (see Fig. S4 in the supplemental material), while the *comFA K152E* mutation does not reduce uptake. NucA, ComEC, and ComFA help to distribute tDNA within the periplasm, and the latter two proteins are certainly also needed for transport to the cytoplasm, while NucA helps with binding as well as uptake. This complexity is consistent with the participation of the proteins in a nanomachine, in which they interact to guide tDNA through the wall, periplasm, and membrane. Further support for this notion is provided by the observations that ComFA stabilizes ComGA (Fig. S1) and that ComFC binds to ComFA (31). Coimmunoprecipitation experiments in B. subtilis have shown that ComGA associates with ComFA, ComFC, ComEC, and the cytoplasmic proteins DprA, SsbB, and RecA (43), all proteins that participate in transformation, providing further support for the concept of a nanomachine.

The results for *ΔcomGA* and *ΔcomC* mutants conform to previous data for B. subtilis and S. pneumoniae obtained using radiolabeled DNA (24, 44, 50), showing that in the absence of these proteins, there is no detectable binding of tDNA to the mutant cells. This reinforces the belief that the tpilus is the initial site of tDNA binding and is consistent with the localization of both ComGA and the initial sites of DNA binding near the poles (47). tpilus retraction has now been observed in both Vibrio cholerae and S. pneumoniae (6, 22) and is very likely to also occur in B. subtilis, although both Gram-positive organisms lack retraction ATPases. Based on several recent studies, it is tempting to conclude that the B. subtilis pilus retracts by spontaneous disassembly into the membrane without an external energy source (6, 51–53). However, a dual-function ATPase has been described that drives both assembly and retraction of a t4 pilus (54), and this remains a possibility for ComGA. In fact, the major effect of *ΔcomFA* on uptake compared to binding (Table 1 and Fig. S4) could be explained by the decreased amount of ComGA (Fig. S2),

In the absence of ComEA, tDNA binding is reduced, and uptake is eliminated. There is experimental evidence for an initially transient form of binding (44), suggesting that attachment of tDNA to the tpilus is reversible. ComEA is too small to extend from the membrane through the wall to aid in the initial binding, and attachment of tDNA to the cell is likely to be stabilized by contact between the tDNA and ComEA in the periplasm. During the uptake of tDNA, no dramatic relocation of the membrane-anchored YFP-ComEA comparable to the reorganization reported for *Vibrio* and *Neisseria* (8, 9, 12) was observed. We propose that inwardly diffusing DNA segments are first captured by fixed or locally mobile ComEA molecules. Further uptake and the spreading of rDNA within the periplasm then results from the inward diffusion of additional segments of DNA, which are trapped by more distant ComEA molecules, in an on-going Brownian ratchet process. The dependence of uptake on NucA and ComEC and the restricted distribution of DNase-resistant rDNA in the *comEC518*, *nucA*, and *comFA K152E* mutants suggests that a more complex mechanism takes place than in the Gram-negative bacteria, where uptake depends only on the tpilus and ComEA. This complexity may be characteristic of bacteria with membrane-anchored, relatively immobile ComEA.

A role for ComEC in uptake to the periplasm, in addition to its undoubted role is transport, was unexpected. The B. subtilis ComEC contains two conserved periplasmic domains that are obvious candidates for involvement in uptake (29, 55, 56): the N-terminal N-loop or OB-domain (PFAM13567) and the C-terminal metallo-β-lactamase domain (PFAM00753) (56). Between the two is the competence domain (PFAM03772) that contains several transmembrane helices and must contribute to forming the transport channel. The restricted localization of rDNA in *comEC* and *comFA* mutants suggests that tDNA is transferred to ComEC in a process that depends on ComFA. The absence of ComEC might arrest uptake by confining the rDNA to locally available ComEA. Transfer to ComEC would also position tDNA for entry to the channel and for degradation of the nontransforming strand. A recent finding for Helicobacter pylori (57) is consistent with these ideas. This organism encodes the unrelated periplasmic DNA binding protein ComH instead of ComEA. ComH interacts directly with the OB-domain of ComEC (57). It has been proposed that the ComEC β-lactamase domain is the nuclease that degrades the nontransforming strand of tDNA (56), and a recent study (58) has confirmed this prediction. If degradation does take place in the periplasm, ComEC may provide an entropic boost to uptake by converting tDNA to single strands and free nucleotides.

The role of NucA in uptake is a novel finding. NucA is anchored in the membrane with its catalytic site protruding into the periplasm where it facilitates uptake (Tables 1 and 2 and Fig. 5), implying that either a nick on one strand of tDNA or a double-strand cut helps with uptake. Indeed, a double-strand cut is likely, as suggested by studies of the NucA paralog, NucB (56) and transformation with circular DNA has shown NucA-dependent conversion to the full-length linear form shortly after binding (37). This result reinforces the idea that tDNA crosses the wall as a folded double-stranded DNA (dsDNA) molecule. Folding DNA, which has a persistence length of about 50 nm (∼147 bp), requires local deformation of the DNA structure, presumably due to binding to the tpilus. After release from the tpilus, strain in the folded tDNA would impel the tDNA to straighten locally and pull back through the wall. A double-strand break induced by NucA would relieve the strain and accelerate the ratchet. An additional explanation for the role of NucA, which is not mutually exclusive with this one, is suggested by the finding that the ComEA protein of V. cholerae prefers binding to a DNA end (8). If this is also true of the B. subtilis protein, NucA might not only relieve the strain in folded tDNA but would also facilitate the initial binding of ComEA to the incoming DNA. These ideas are consistent with our finding that both binding and uptake are reduced in the *nucA* mutants. The termini produced by NucA would also be available to enter the ComEC channel (37).

The results presented in this study allow us to propose an updated model for transformation in B. subtilis, aspects of which are summarized in Fig. 1. First, tDNA binds reversibly to the tpilus, which retracts, bringing a folded loop of DNA into the periplasm. The S. pneumoniae tpilus is 6 nm thick, and retraction would likely leave a hole in the wall of the same size. A folded DNA molecule would have a thickness of 4 nm, permitting the tpilus to bring the DNA into the periplasm with its hydration shell intact, allowing subsequent inward diffusion of the DNA. NucA then cuts the tDNA as it is released from the tpilus, relieving strain introduced by tDNA folding and possibly facilitating binding to ComEA at a terminus. This binding stabilizes the association of tDNA with the cell and as successive segments of DNA diffuse into the periplasm through the cell wall, they are captured by additional ComEA molecules and prevented from diffusing outward. As the ComEA binding sites near the locus of entry become occupied, tDNA spreads in a process dependent on ComFA and ComEC and is captured by ComEC for transport to the cytoplasm, accomplished by ComEC and the ComFA/ComFC complex, which comprise the transformation permease.

The use of labeled DNA that cannot cross the membrane may obscure what may be a seamless process of uptake and transport. In other words, transformation may not be a two-step process in which tDNA accumulates in the periplasm. Another open question concerns the extent to which our revised understanding of DNase resistance applies to other Gram-positive transformable bacteria, such as S. pneumoniae. Despite the shared requirements for ComEA, ComEC, and t4 pilus proteins, there are likely to be important mechanistic differences between the uptake mechanisms of these two model Gram-positive bacteria. In S. pneumoniae, degradation of the nontransforming strand is accomplished by EndA (59, 60) and degradation takes place in the absence of ComEC (38), implying that the latter is not needed for uptake to the periplasm. In contrast, B. subtilis does not encode EndA, and when *comEC* is inactivated, degradation ceases (37), consistent with the role of ComEC in uptake and its action as a nuclease. Finally, in B. subtilis, the tpilus is probably short, while in S. pneumoniae, it extends from the cell surface (20, 23). Many outstanding questions remain, most importantly, how these various processes are accomplished on the molecular level.

## MATERIALS AND METHODS

### Strains and growth conditions.

All mutant strains (see Table S1 in the supplemental material) of B. subtilis were constructed in strain IS75 (*his leu met*) and are derivatives of the domesticated strain 168. All the strains expressed YFP or CFP controlled by the *comG* promoter, inserted ectopically in *amyE*. B. subtilis was grown to competence for all experiments by the two-step method (61) except that unfrozen cultures were used and cultures were started from plates grown overnight instead of from spore suspensions.

10.1128/mBio.01061-21.1 TABLE S1 Strains used in this study. Download Table S1, DOCX file, 0.02 MB. Copyright © 2021 Hahn et al. 2021 Hahn et al. https://creativecommons.org/licenses/by/4.0/ This content is distributed under the terms of the Creative Commons Attribution 4.0 International license.

### Transformation for microscopy.

Competent cultures of P_G_-CFP- and P_G_-YFP-expressing mutant and wild-type strains were mixed 1:1, and the mixture was incubated with 0.2 μg/ml of rhodamine-labeled DNA for the indicated times, in final volumes of 300 to 500 μl. Samples were taken and either immediately fixed by the addition of 3.2% paraformaldehyde and incubation at room temperature for 30 min or were first incubated for 3 min with 100 μg/ml DNase I at 37°C before fixation. In the experiment shown in Fig. 2, where DNase-treated and untreated samples were combined for visualization, the cells were centrifuged briefly and resuspended in the same medium used to achieve competence but containing no Mg and EDTA (10 mM) to stop the nuclease activity before mixing and fixation. Samples were then washed and imaged.

### Preparation of rDNA.

Bacteriophage λ DNA (New England Biolabs) was diluted to 10 μg/ml and incubated for 60 min with a Mirus Label-IT rhodamine TM reagent (Mirus Bio) according to the manufacturer’s recommendations. The DNA was then processed through a spin column to remove excess reagent.

### Point mutation of *nucA*.

A fragment of DNA carrying the *nucA* sequence with a D98A mutation and BamHI ends was synthesized by Biomatik (Canada), and cloned into the BamHI site of pMiniMAD2, a gift from Dan Kearns (Indiana University). After transformation with selection for erythromycin (5 μg/ml), the markerless chromosomal mutant was isolated as described by Patrick and Kearns (62).

### YFP-ComEA construction.

Three fragments were produced by PCR and assembled using NEBuilder HiFi into pUC18CM, a derivative of pUC18 with a chloramphenicol resistance cassette for selection in B. subtilis. Fragment 1 carried 1,500 bp upstream from the *comEA* coding sequence, including the promoter, the ribosomal binding site, and start codon. To produce this fragment, primers 1 and 2 were used (Table S2). Fragment 2 carried the *yfp* open reading frame with the stop codon omitted, produced using primers 3 and 4. Fragment 3 consisted of the *comEA* open reading frame without its start codon, produced using primers 5 and 6. After assembly, the clone was verified by sequencing and transformed into the native locus via a single-crossover event. The duplicated native *comEA* open reading frame was then deleted by transformation using DNA from strain BD8739 (*comEA*::*ery trpC2*), and the final construction was again verified by sequencing.

10.1128/mBio.01061-21.2 TABLE S2 Primers used in this study. Download Table S2, DOCX file, 0.01 MB. Copyright © 2021 Hahn et al. 2021 Hahn et al. https://creativecommons.org/licenses/by/4.0/ This content is distributed under the terms of the Creative Commons Attribution 4.0 International license.

### Microscopy.

For microscopy, 1-μl samples of transformed cells were placed on thin agarose pads before visualization. All images were acquired with a 100× Plan Apo immersion objective, numerical aperture (NA) of 1.40 on a Nikon T*i* microscope, equipped with light-emitting diode (LED) excitation sources and an Orca Flash 4.0 camera (Hamamatsu). Nikon Elements was used for data acquisition and image analysis. Z-stack images were recorded with 200-nm steps and processed for 3D deconvolution with the Landweber algorithm in Nikon Elements. Volume reconstructions were assembled with alpha blending. Images were exported to Adobe Photoshop and Microsoft PowerPoint for final processing.

### Western blotting.

Western blotting was carried out using standard methods with semidry blotting, and the nitrocellulose blots were developed using ECL (GE Healthcare). The images were recorded with a Bio-Rad ChemiDoc MP imager. In most gels, for a loading control, the top of the membrane was cut off after blotting and developed separately with anti-elongation factor G (anti-EFG) antiserum, a kind gift from Jonathan Dworkin (Columbia University Medical School).

### Measurement of rDNA fluorescence intensity.

RGB images were split, and the rDNA channels were imported into Image J Fiji (63, 64), where they were converted to 16-bit gray scale images. Background pixel intensities, measured from areas without cells, were subtracted from all the pixels in each field, and the integrated densities of individual wild-type and mutant cells were measured within fields. To combine data across fields for a given mutant, the average intensity of wild-type cells was computed in each field to obtain normalizing factors that were applied to all the cells (wild type and mutant) so that the averages for wild-type cells were the same in each field.

### Statistical analysis.

The data in Table 1 were analyzed using a two-tailed z-test. The *P* values in Table 2 were obtained using both a two-tailed *t* test and a Mann-Whitney U-test, which reported that the distributions were approximately normal.

### Data availability.

The data that support the findings of this study are available from the corresponding author (D. Dubnau) upon request.
