# Supplementary figures and images for: Mechanisms of Transforming DNA Uptake to the Periplasm of Bacillus subtilis
Source: mBio. 2021 Jun 15;12(3):e01061-21. doi: 10.1128/mBio.01061-21 (PMC8262900; doi:10.1128/mBio.01061-21)

Volume reconstructions of wild-type cells after DNase treatment, without fixation

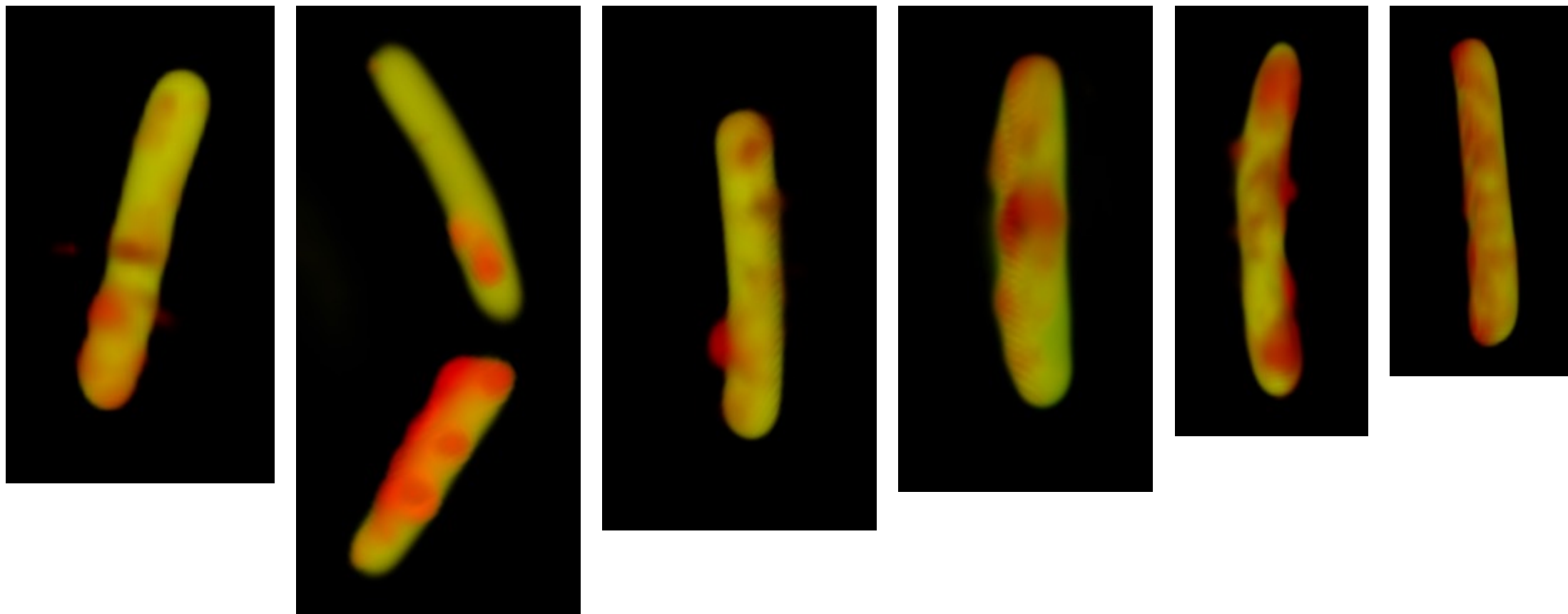

Supplement: FIG S1 [file mbio.01061-21-sf001.pdf]

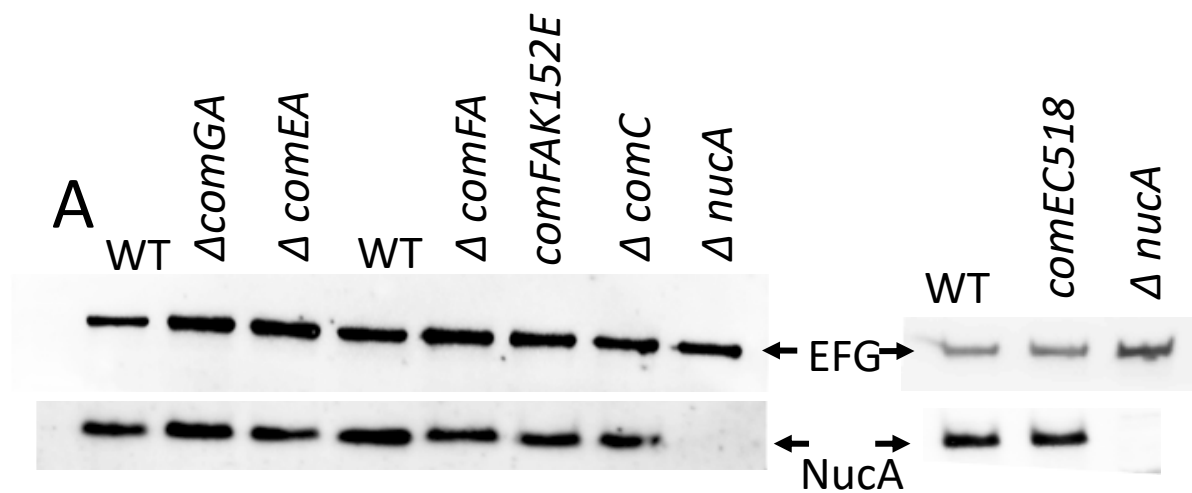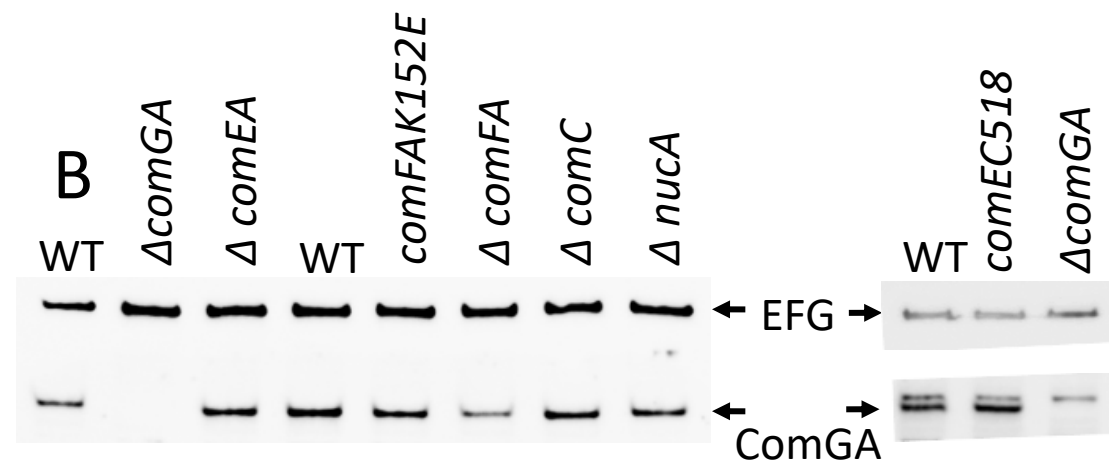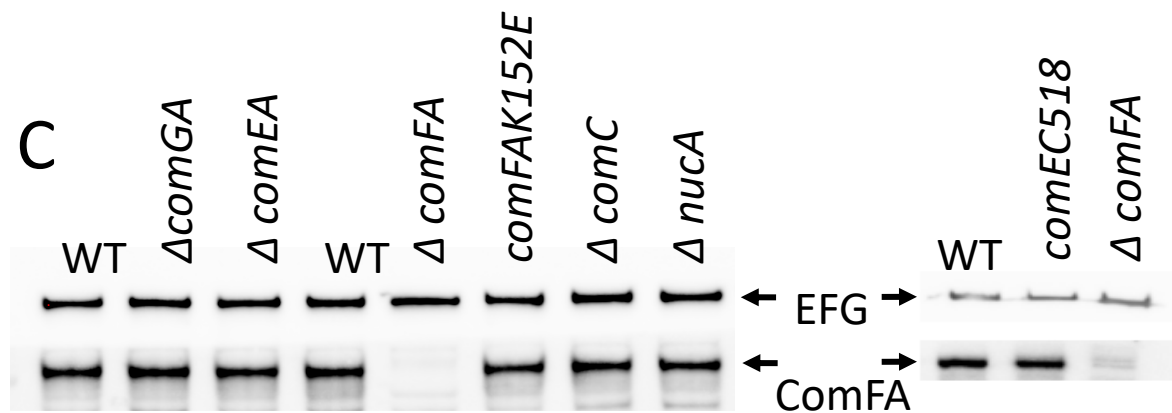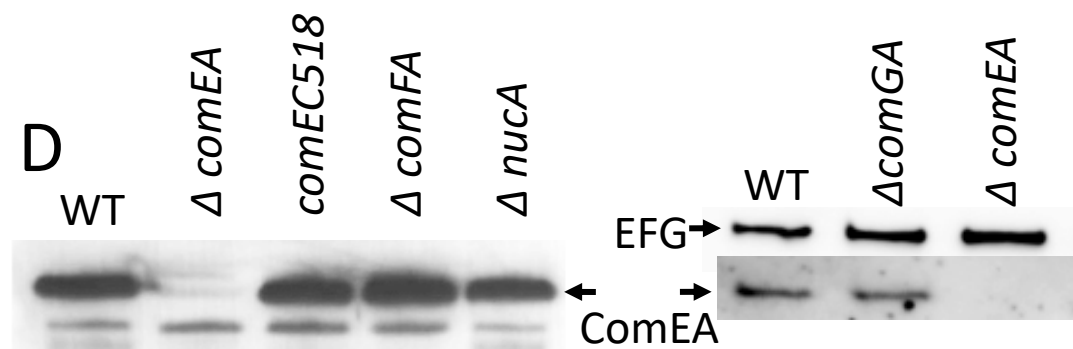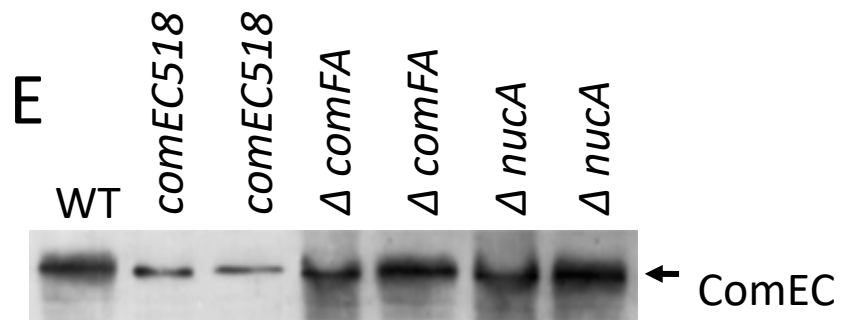

Supplement: FIG S2 [file mbio.01061-21-sf002.pdf]

Wild-type (YFP) and  $\Delta comGA$  (CFP) strains

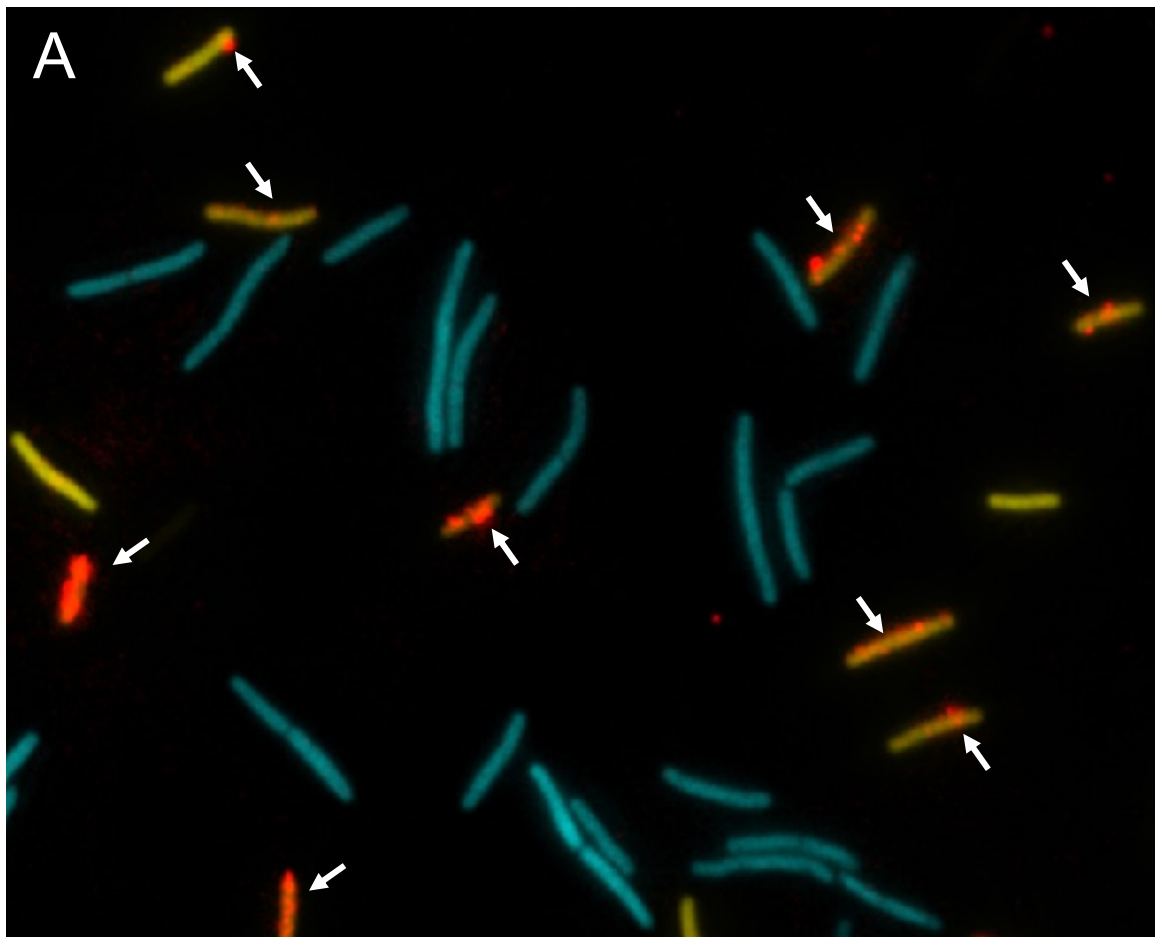

Wild-type (YFP) and  $\Delta comC$  (CFP) strains

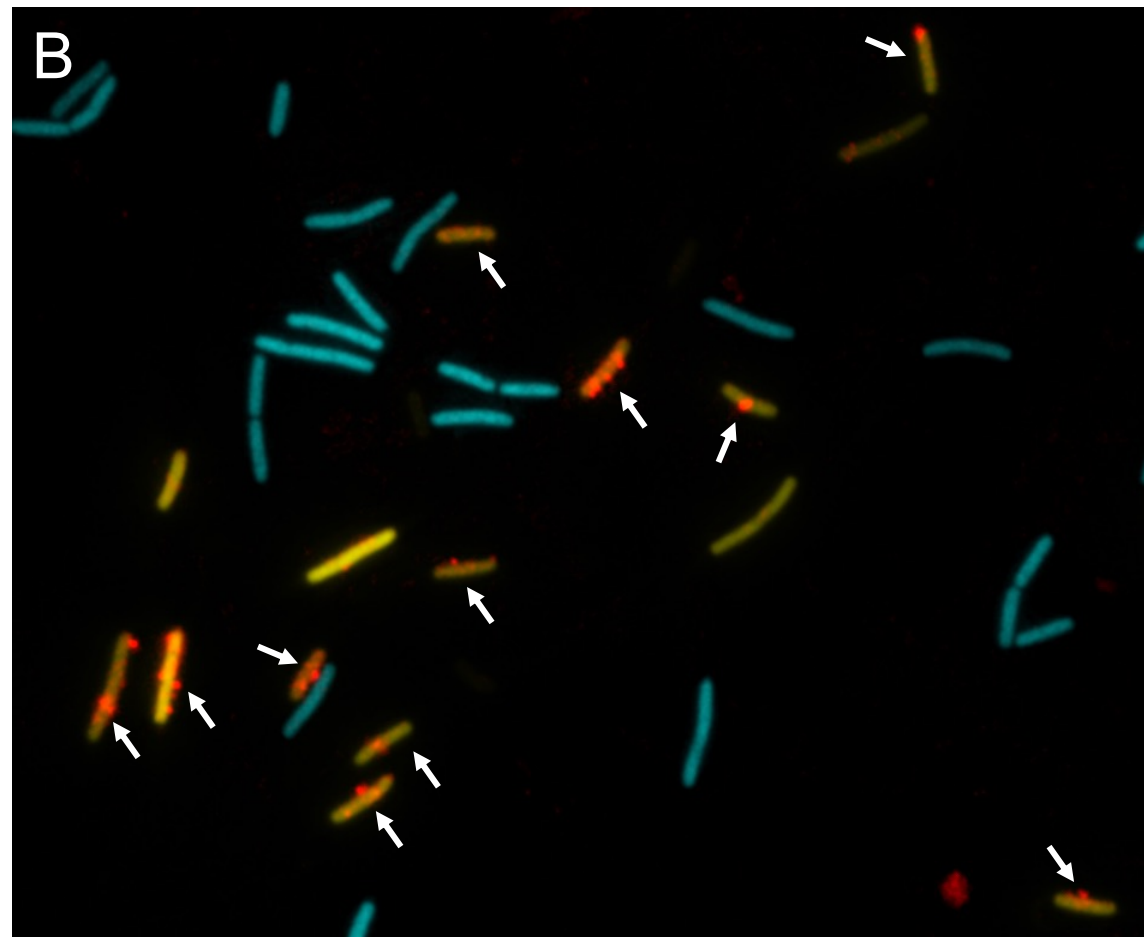

Supplement: FIG S3 [file mbio.01061-21-sf003.pdf]

Wild-type (YFP) and  $\Delta comFA$  (CFP) cells

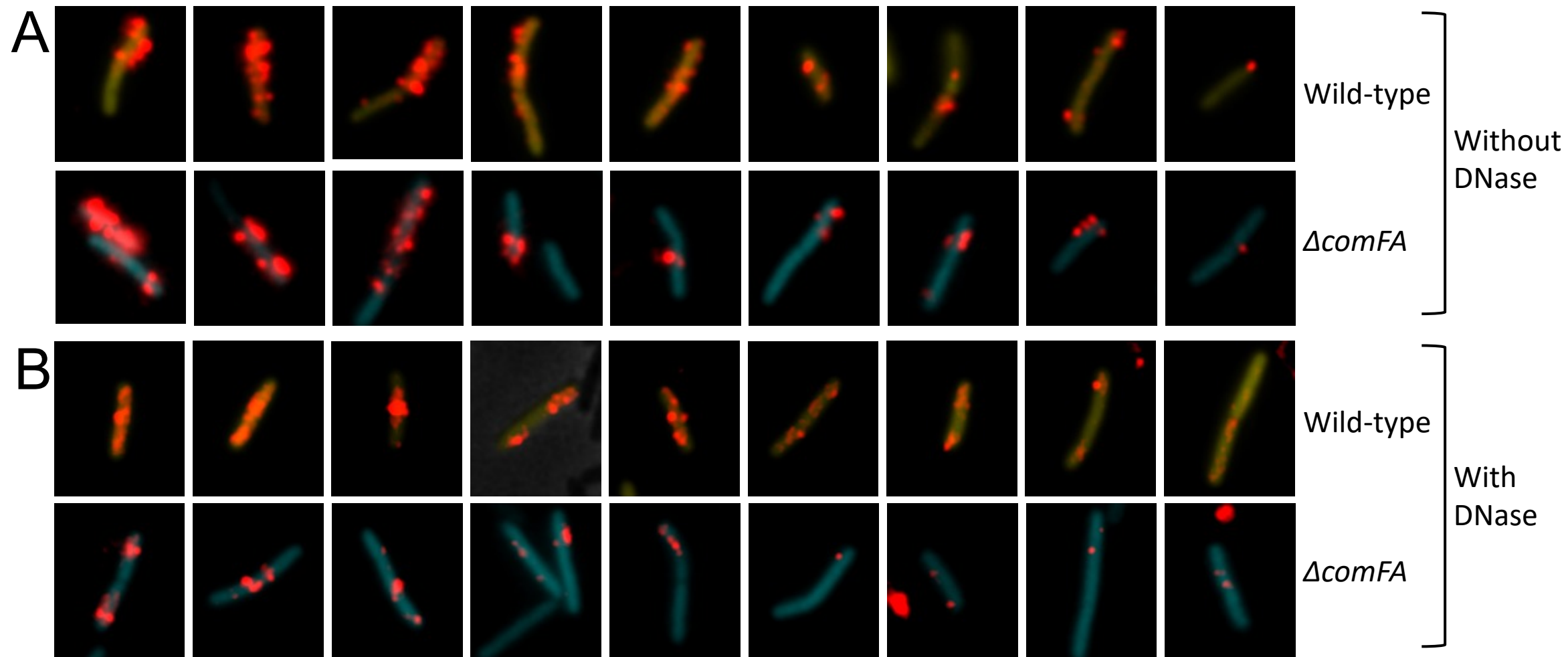

Supplement: FIG S4 [file mbio.01061-21-sf004.pdf]

Wild-type (YFP) and  $\Delta nucA$  (CFP) strains

A

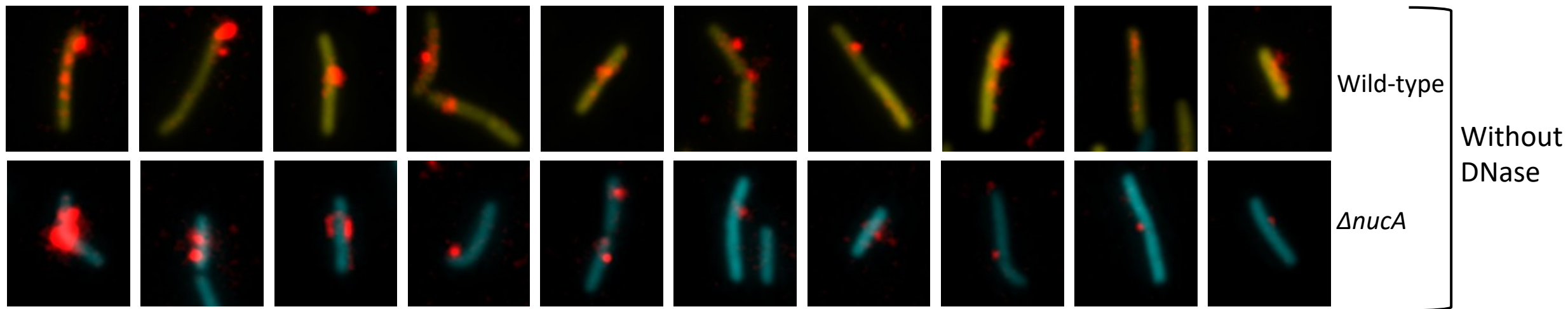

B

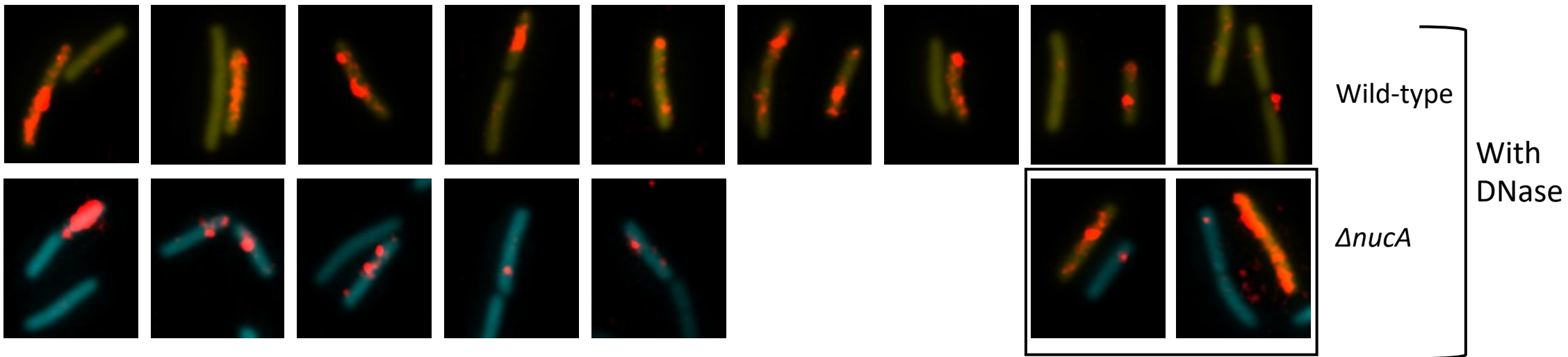

Supplement: FIG S5 [file mbio.01061-21-sf005.pdf]

# Wildtype, $\Delta nucA$ and $nucAD98A$ volume reconstructions

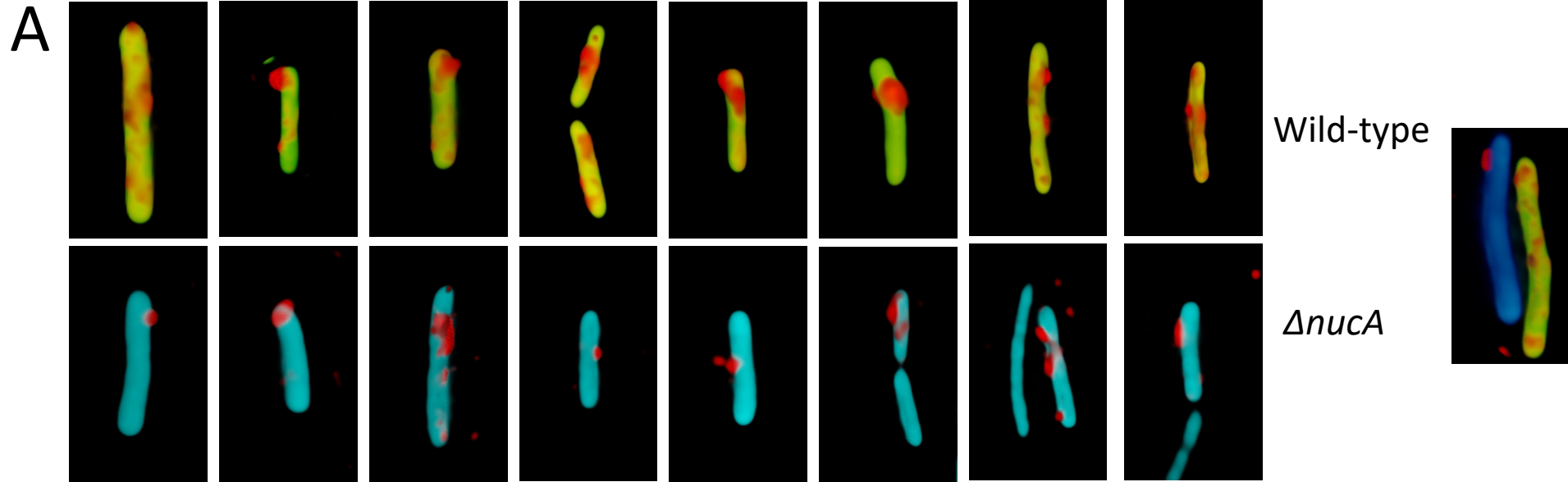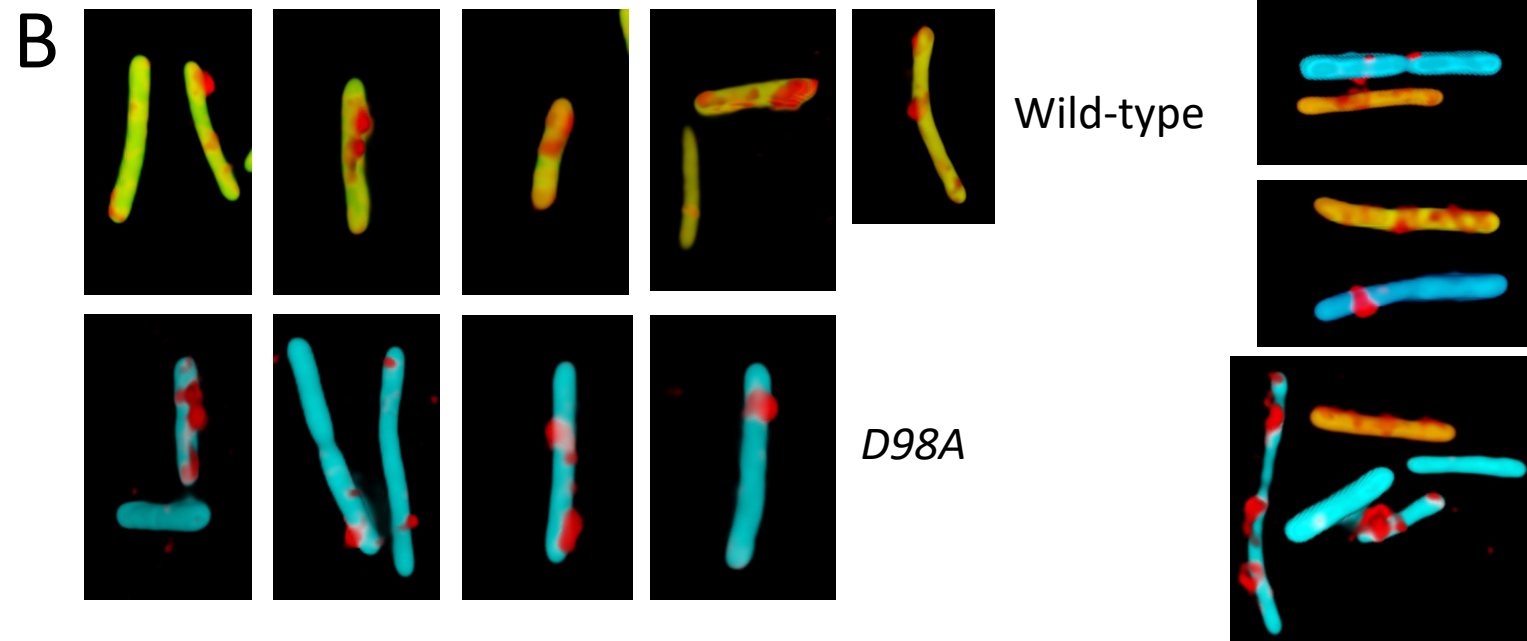

Supplement: FIG S6 [file mbio.01061-21-sf006.pdf]
