# Supplementary material for: Mechanisms of Transforming DNA Uptake to the Periplasm of Bacillus subtilis
Source: mBio. 2021 Jun 15;12(3):e01061-21. doi: 10.1128/mBio.01061-21 (PMC8262900; doi:10.1128/mBio.01061-21)
Supplement: TABLE S1 [file mbio.01061-21-st001.docx]

Table S1

Strains

| Strain^a^ | Genotype | Source |
| --- | --- | --- |
| BD5810 | *amyE::P_G-_CFP (spc)* | (48) |
| BD6011 | *amyE::P_G-_YFP (spc)* | (48) |
| BD8739 | t*rpC2 ΔcomEA (ery)* *^b^* |  |
| BD8800 | *amyE::Pr_G-_CFP (spc) ΔcomGA(ery)^b^* | This work |
| BD8802 | *amyE:: Pr_G-_CFP (spc) ΔcomEA(ery) ^b^* | This work |
| BD8805 | *amyE::P_G-_CFP (spc) ΔnucA(ery) ^b^* | This work |
| BD8883 | *amyE::P_G-_CFP (spc) ΔcomC (ery) ^b^* | This work |
| BD8884 | *amyE::P_G-_CFP (spc) comFA K152E* | (33) |
| BD8911 | *amyE::P_G-_CFP (spc) ΔcomFA (ery) ^b^* | This work |
| BD8949 | *amyE::P_G-_CFP (spc) yfp-comEA ΔcomEA (ery) ^b^* | This work |
| BD8951 | *amyE::P_G-_CFP (spc) nucAD98A* | This work |
| BD8954 | *amyE::P_G-_CFP (spc) comEC-518* | (29) |

^a^All of the listed strains except BD8739 were derivatives of IS75 *his leu met*.

*^b^*These deletion mutations, constructed by Koo et al (63), were obtained from the Bacillus Genetic Stock Center
