# Supplementary material for: Mechanisms of Transforming DNA Uptake to the Periplasm of Bacillus subtilis
Source: mBio. 2021 Jun 15;12(3):e01061-21. doi: 10.1128/mBio.01061-21 (PMC8262900; doi:10.1128/mBio.01061-21)
Supplement: TABLE S2 [file mbio.01061-21-st002.docx]

Table S2

Primers

| **Primer number** | **Sequence (5’->3’)** |
| --- | --- |
| 1 | AGAGGATCCCCGGGTACCGAGCTCGAATTCAAAAGGTATCGAGTAGCCAG |
| 2 | CTCCTTTACTCATATTCATCGTGCATGTTCC |
| 3 | ATGCACGATGAATATGAGTAAAGGAGAAGAAC |
| 4 | TCAACCAATTCATTTTGTATAGTTCATCCATGC |
| 5 | GGAAACAGCTATGACCATGATTACGAATTCACTTTACTGTAATGGAAG |
| 6 | ACTATACAAATAAATGAATTGGTTGAATCAGC |
